# Genomic and Proteomic Analyses of the Fungus *Arthrobotrys oligospora* Provide Insights into Nematode-Trap Formation

**DOI:** 10.1371/journal.ppat.1002179

**Published:** 2011-09-01

**Authors:** Jinkui Yang, Lei Wang, Xinglai Ji, Yun Feng, Xiaomin Li, Chenggang Zou, Jianping Xu, Yan Ren, Qili Mi, Junli Wu, Shuqun Liu, Yu Liu, Xiaowei Huang, Haiyan Wang, Xuemei Niu, Juan Li, Lianming Liang, Yanlu Luo, Kaifang Ji, Wei Zhou, Zefen Yu, Guohong Li, Yajun Liu, Lei Li, Min Qiao, Lu Feng, Ke-Qin Zhang

**Affiliations:** 1 Laboratory for Conservation and Utilization of Bio-Resources, and Key Laboratory of Microbial Diversity in Southwest China, Ministry of Education, Yunnan University, Kunming, P. R. China; 2 TEDA School of Biological Sciences and Biotechnology, Nankai University, Tianjin, P. R. China; 3 Department of Biology, McMaster University, Hamilton, Ontario, Canada; 4 Yunnan Academy of Tobacco Science, Kunming, P. R. China; University of Melbourne, Australia

## Abstract

Nematode-trapping fungi are “carnivorous” and attack their hosts using specialized trapping devices. The morphological development of these traps is the key indicator of their switch from saprophytic to predacious lifestyles. Here, the genome of the nematode-trapping fungus *Arthrobotrys oligospora* Fres. (ATCC24927) was reported. The genome contains 40.07 Mb assembled sequence with 11,479 predicted genes. Comparative analysis showed that *A. oligospora* shared many more genes with pathogenic fungi than with non-pathogenic fungi. Specifically, compared to several sequenced ascomycete fungi, the *A. oligospora* genome has a larger number of pathogenicity-related genes in the subtilisin, cellulase, cellobiohydrolase, and pectinesterase gene families. Searching against the pathogen-host interaction gene database identified 398 homologous genes involved in pathogenicity in other fungi. The analysis of repetitive sequences provided evidence for repeat-induced point mutations in *A. oligospora*. Proteomic and quantitative PCR (qPCR) analyses revealed that 90 genes were significantly up-regulated at the early stage of trap-formation by nematode extracts and most of these genes were involved in translation, amino acid metabolism, carbohydrate metabolism, cell wall and membrane biogenesis. Based on the combined genomic, proteomic and qPCR data, a model for the formation of nematode trapping device in this fungus was proposed. In this model, multiple fungal signal transduction pathways are activated by its nematode prey to further regulate downstream genes associated with diverse cellular processes such as energy metabolism, biosynthesis of the cell wall and adhesive proteins, cell division, glycerol accumulation and peroxisome biogenesis. This study will facilitate the identification of pathogenicity-related genes and provide a broad foundation for understanding the molecular and evolutionary mechanisms underlying fungi-nematodes interactions.

## Author Summary

The fungus *Arthrobotrys oligospora* has multiple lifestyles. It's not only a nematode pathogen, but also a saprophyte, a pathogen of other fungi, and a colonizer of plant roots. As a nematode pathogen, *A. oligospora* forms adhesive networks to capture nematodes and is a model organism for understanding the interaction between these fungi and their host nematodes. In this study, the whole genome sequence of *A. oligospora* was reported. Our analyses of the proteome profiles of intracellular proteins from cells treated with nematode extracts for 10 h and 48 h revealed a key set of genes involved in trap formation. The changes in protein levels for some trap formation related genes were further confirmed by qPCR. The combined genome and proteome analysis identified the major genetic and metabolic pathways involved in trap formation in *A. oligospora*. Our results provide the first glimpse into the genome and proteome of this fascinating group of carnivorous fungi. The data should serve as a roadmap for further investigations into the interaction between nematode-trapping fungi and their host nematodes, providing broad foundations for research on the biocontrol of pathogenic nematodes.

## Introduction

Nematode-trapping fungi are a heterogeneous group of organisms broadly distributed in terrestrial and aquatic ecosystems [1], [2]. These fungi are capable of developing specific trapping devices such as adhesive networks, adhesive knobs, and constricting rings to capture nematodes and then extract nutrients from their nematode prey [2], [3], [4]. Most nematode-trapping fungi can live as both saprophytes and parasites [1], [2]. They play important roles in maintaining nematode population density in diverse natural environments. Their broad adaptability and flexible lifestyles also make them ideal agents for controlling parasitic nematodes of plants and animals [2], [4].

**Figure 1:**
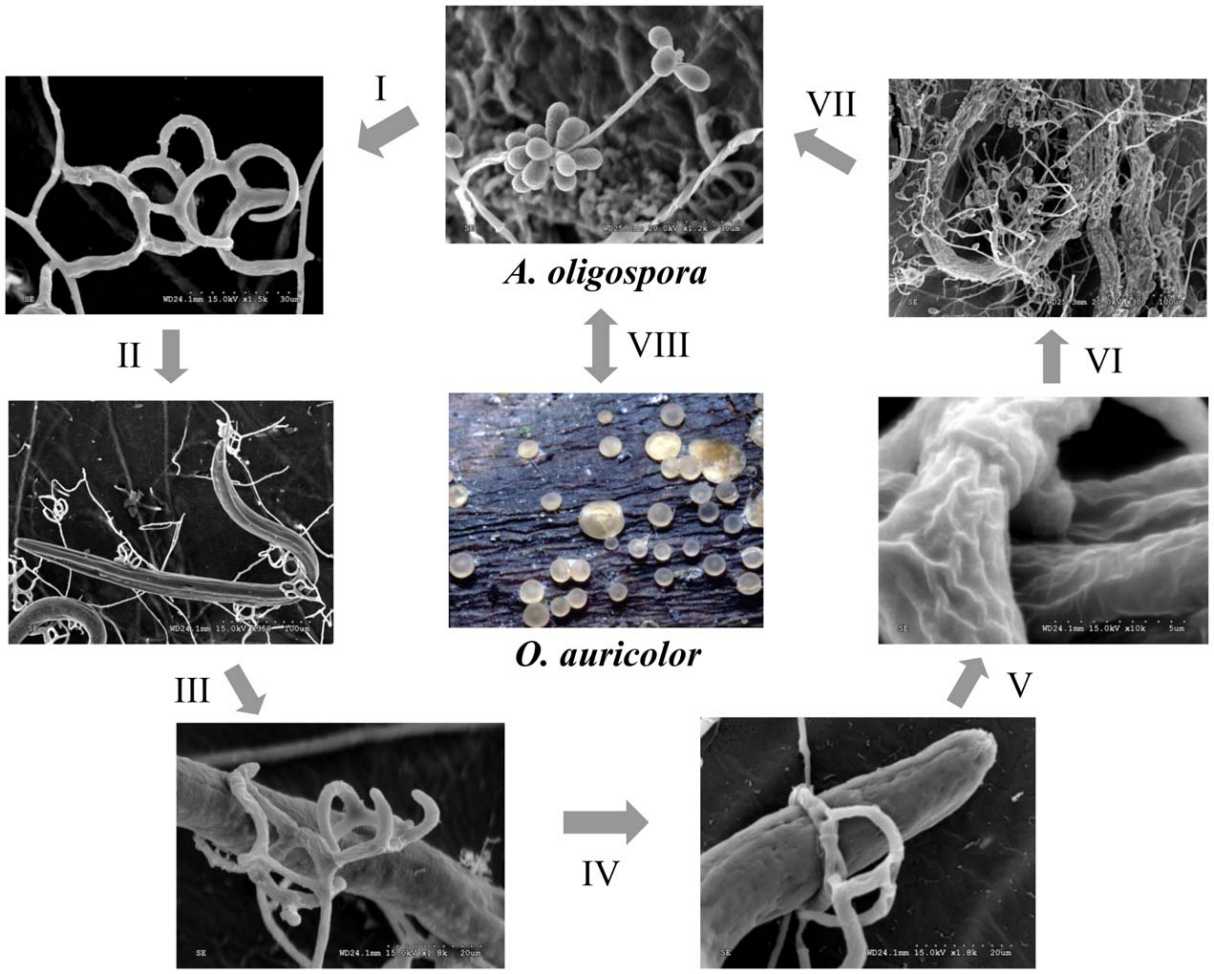
Saprophytic and parasitic stages of the nematode-trapping fungus *A. oligospora*. The life cycle of *A. oligospora* includes three stages: saprophytic stage, transitional stage, and parasitic stage. The parasitic stage can be divided into the following six steps: I. Trap formation; II. Attraction; III. Adhesion; IV. Capturing; V, Penetration and immobilization; and VI. Digestion and assimilation. The saprophytic stage (VII) and sexual stage (VIII) are also shown.

*Arthrobotrys oligospora* (teleomorph *Orbilia auricolor*) is one of the best-studied nematode-trapping fungi [2], [5]. Strains of *A. oligospora* have been found in diverse soil environments including heavy metal-polluted soils and decaying wood [6], [7] where they live mainly as saprophytes. In the presence of nematodes, *A. oligospora* enters the parasitic stage by forming complex three-dimensional networks to trap nematodes (Figure 1). The trapping initiates a series of processes including adhesion, penetration, and immobilization of nematodes [2], [5]. The ability to trap nematodes makes it an attractive candidate agent for controlling parasitic nematodes of plants and animals. Indeed, two commercial biological nematicides, Royal 300 [8] and Royal 350 [9], have been developed based on two species closely related to *A. oligospora*: *A. robusta* and *A. irregularis*.

The formation of trapping devices by nematode-trapping fungi is an important indicator of their switch from the saprophytic to the predacious lifestyles [2], [4]. Previous studies have identified the morphological characteristics of major trapping devices, including their evolution and phylogenetic distribution [4], [10], [11], [12]. Recently, the gene expression profiles in trap cells and vegetative hyphae of the nematode-trapping fungus *Monacrosporium haptotylum* were analyzed using the microarray technology [13], including examining the transcriptional response of *M. haptotylum* to the model nematode *Caenorhabditis elegans* at different infection stages [14]. However, at present, no genome sequence information is available in any nematode-trapping fungi and relatively little is known about the molecular mechanism underlying trap formation or nematode-fungi interaction. Such genome sequence information could help reveal the genetic features that allow these fungi to form nematode traps and provide important information to study the molecular mechanism of infection and their lifestyle transitions.

Here the first genome sequence of a nematode-trapping fungus, *A. oligospora* Fres. (ATCC24927) was reported. In addition, using the proteomics approach and quantitative PCR (qPCR), proteins differentially expressed in response to nematode extract (NE) were identified and further investigated. The combined genomic, proteomic, and qPCR data led us to formulate the putative genetic and metabolic pathways involved in trap formation in *A. oligospora*.

## Results

### Genome sequencing and analysis

**Figure 2:**
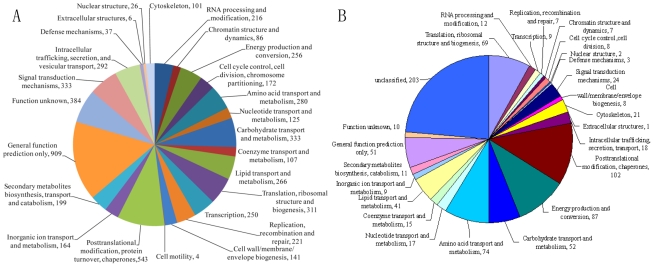
Annotation of the *A. oligospora* genome and proteome by COG/KOG categories. The proteins were assigned into different KOG/COG functionary categories as shown in the pie chart. A. A total of 5762 proteins from *A. oligospora* genome were annotated by KOG/COG. B. Classification of the *A. oligospora* intracellular proteins detected by 2-DE. A total of 861 proteins were identified from *A. oligospora* cells treated with and without NE for 10 h and 48 h respectively.

The genome of *A. oligospora* strain ATCC24927 was sequenced to 36.6-fold coverage through a Sanger/pyrosequencing hybrid shotgun approach from multiple clone types (Table S1). The 40.07 Mb genome is similar in size to that of the model ascomycete fungus *Neurospora crassa* [15]. The *A. oligospora* genome was assembled into 215 scaffolds, containing a long-range continuity as reflected by N50 scaffold size of 2037 Kb and N50 contig size of 575.8 Kb (Table S2). The assembly represents 99% of the coding regions of the genome, as assessed by mapping 50,121 assembled transcript sequences (8.9 Mb) to the genome assembly. Almost all of the transcripts (99.6%) were mapped onto the genome. A total of 11,479 protein-coding genes were predicted: 23.6% belonged to multi-gene families and 44.6% were mapped in the KOG/COG database (Figure 2A). The average gene density was one gene per 3.50 kb, with an average gene length of 1.69 kb, similar to that of *N. crassa* (1.67 kb) [15]. Compared to *N. crassa*, genes in *A. oligospora* have more introns (2.8 VS. 1.7) but with a shorter average intron length (90 bp VS. 134 bp) [15]. As is typical of ascomycete fungal genomes, approximately one-third (34.6%) of the predicted *A. oligospora* genes lacked significant homologies to known proteins from public databases (Table S2).

### Comparative genomic analysis

**Figure 3:**
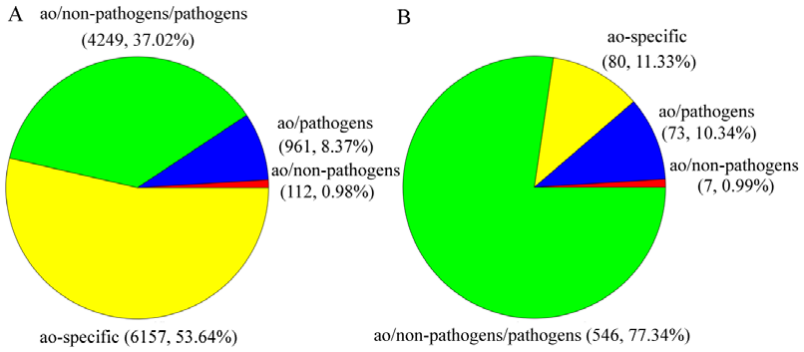
The distributions of *A. oligospora* genes and PHI putative genes in different categories. The other 10 fungal genomes were divided into non-pathogen and pathogen genomes. Based on orthology analysis, the genes in *A. oligospora* were classified into four categories: ao-specific (only found in *A. oligospora*), ao/pathogen (also found in pathogenic fungi), ao/pathogen/non-pathogen (found in all genomes) and ao/non-pathogen (also found in non-pathogenic fungi). A. The distribution of all *A. oligospora* genes in different categories. B. The distribution of PHI putative genes in different categories.

The orthologous genes between *A. oligospora* and other 10 fungal genomes were identified based on bidirectional best hits (BBHs) using BLAST [16] (Table S3). The other 10 genomes used for comparison were divided into non-pathogen (*Aspergillus nidulans*, *N. crassa* and *Saccharomyces cerevisiae*) and pathogen (*Fusarium graminearum*, *Magnaporthe oryzae*, *Verticillium dahliae*, *A. fumigatus*, *Coccidioides immitis*, *Histoplasma capsulatum* and *Chaetomium globosum*) groups. Based on orthology analysis, the genes in *A. oligospora* can be classified into four categories: ao (only found in *A. oligospora*, 6157 genes), ao/pathogen (found in *A. oligospora* and pathogen genomes, 961 genes), ao/pathogen/non-pathogen (found in all genomes, 4249 genes), and ao/non-pathogen (found in *A. oligospora* and non-pathogen genomes, 112 genes) (Figure 3A). The results showed that *A. oligospora* shared many more genes with pathogenic fungi than with non-pathogenic fungi. The genes shared between *A. oligospora* and other pathogenic fungi may be functionally related to pathogenicity in these fungi.

### Genome evolution

**Figure 4:**
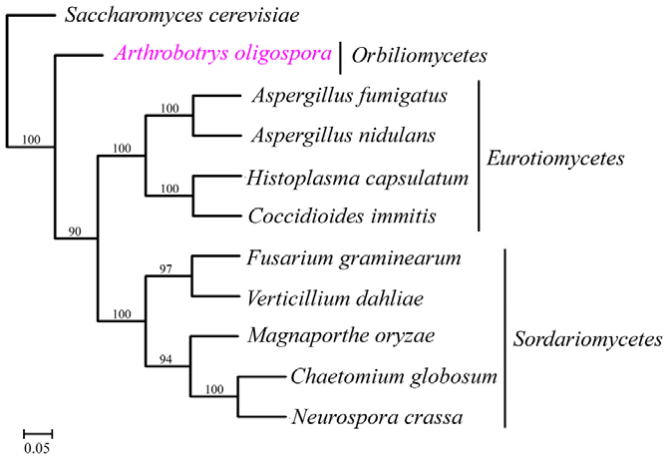
The phylogenomic tree was constructed based on orthologous proteins from 11 fungal genomes using Maximum likelihood method.

A total of 529 orthologous proteins were found in all the 11 analyzed fungal genomes. These orthologous sequences were concatenated to infer phylogenomic relationships among these fungi using Neighbor-joining, Maximum parsimony and Maximum likelihood methods. The same tree topology was found by all the three phylogenetic methods (Figure 4). From the phylogenomic trees, Eurotiomycete fungi (*A. nidulans*, *A. fumigatus*, *C. immitis* and *H. capsulatum*) were clustered into one clade, Sordariomycete fungi (*F. graminearum*, *V. dahliae*, *C. globosum*, *N. crassa* and *M. grisea*) were clustered into a different clade, while *A. oligospora* formed a separate branch. Our result was consistent with results from previous phylogenomic analyses of the Ascomycota [17], [18].

Our analysis revealed that 0.47% of the assembly consisted of repetitive sequences (Table S4), higher than that in the ascomycete wheat pathogen *F. graminearum* (0.10%) [19], but lower than that in *N. crassa* (3.15%) [15]. The relatively low percentage of repetitive sequences in *N. crassa* was attributed to a genome-wide defense system known as repeat-induced point mutation (RIP), a phenomenon hypothesized to be widespread among filamentous ascomycetes [15], [19]. To determine whether RIP contributed to the low percentage of repetitive sequences in *A. oligospora*, the RIP indices for the whole genome as well as separately for the coding regions, non-coding regions, exons, introns, multigene families, and repetitive sequences were calculated. Using the default setting (TpA/ApT≥0.89, (CpT+ApT)/(ApC+GpT) ≤1.03) [20], a positive RIP response was detected in the repetitive sequences with both RIP indices (and very high AT content) (Table S5), likely contributing to the lack of the repetitive sequences in *A. oligospora*. RIP could have similarly contributed to the relatively low proportion of genes in multigene families (25.11%) in *A. oligospora*. For protein coding genes, although no positive RIP responses were detected using average RIP indices, a large number of regions contained signatures of RIP (Table S6). Specifically, using the 200-bp windows with 100-bp shifts, about one third of the genome sequences had RIP-positive sequences.

### Pathogenicity

The whole genome blast analysis against the pathogen-host interaction (PHI) gene database [21] identified 398 putative PHI genes in *A. oligospora*, 294 of which belong to 86 multigene families. The gene number expanded to 706 if single linkage transitive closure was applied. The distribution analysis of the othologous sequences showed that the putative PHI genes included 80 ao-specific genes and 73 genes only found in pathogen genomes, more than those in the ao/non-pathogen category (Figure 3B). It should be noted that many putative PHI genes were not included in the pathogenicity-related gene families, suggesting that there are potentially more pathogenicity-related genes that remain to be experimentally confirmed.

**Figure 5:**
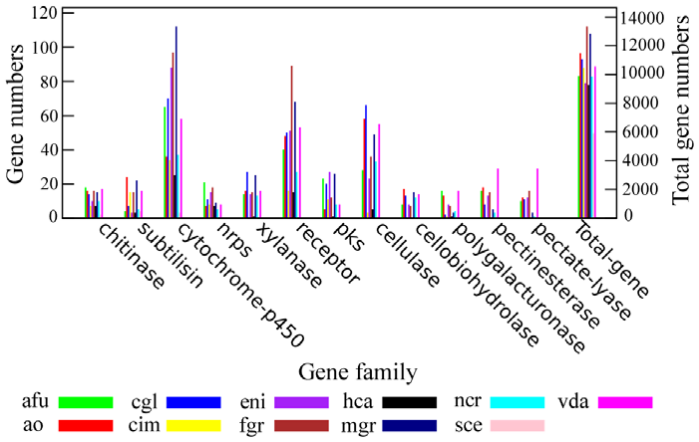
Comparison of pathogenicity-related gene families between *A. oligospora* and other sequenced fungi. Several pathogenicity-related enzyme families were compared between *A. oligospora* and other 10 sequenced fungi including *A. fumigatus* (afu), *C. globosum* (cgl), *C. immitis* (cim), *A. nidulans* (eni), *F. graminearum* (fgr), *H. capsulatum* (hca), *M. grisea* (mgr), *N. crassa* (ncr), *S. cerevisiae* (sce) and *V. dahliae* (vda).

The number of genes in several gene families related to fungal pathogenicity was found expanded in the *A. oligospora* genome (Figure 5). For example, subtilisins, a group of proteases essential for infection [22], [23], [24], [25], were found in a greater number in *A. oligospora* (24) than in several model ascomycetes such as the animal pathogens *A. fumigatus* (4), *C. immitis* (15) and *H. capsulatum* (3), plant pathogens *F. graminearum* (15), *V. dahliae* (16) and *M. grisea* (22), and non-pathogens *A. nidulans* (3), *N. crassa* (5). Besides subtilisin genes, the *A. oligospora* genome also contains a larger number of genes within enzyme families of cellulases (58), pectinesterases (18) and cellobiohydrolases (17) than other sequenced model fungi (Figure 5). As a comparison, the *M. grisea* genome contains the highest number of genes within the cytochrome P450 family (P450; 112); the *A. fumigatus* genome contains the highest numbers of genes within enzyme families of non-ribosomal peptide synthases (NRPS; 21), chitinases (18), and polygalacturonases (16); the *V. dahliae* genome contains the highest numbers of genes within enzyme families of polygalacturonases (16), pectinesterases (29), and pectate lyases (29); and the *C. globosum* genome contains the highest numbers of genes within enzyme families of cellulases (66) and xylanases (27).

Because of their ability to disrupt the physical and physiological integrity of the cuticles of nematode hosts during penetration and colonization, subtilisin-like serine proteases have been identified as important virulence factors in nematode-trapping fungi [23], [26], [27]. For example, previous studies demonstrated that a proteinase K-like serine protease PII in *A. oligospora* could efficiently degrade nematode cuticle [22], [26], [28]. The *A. oligospora* genome contains 24 genes encoding putative subtilases, which can be categorized into four subtilisin families (Figure S1). Among them, 20 belong to the proteinase K-like family, which can be further classified into five subfamilies (SF1-SF5, Figure S1). The following four genes are clustered into one of the five sub-families, SF4: *PII* (AOL_s00076g4), *P186* (AOL_s00215g702), *P233* (AOL_s00075g8), and *P12* (AOL_s00170g103). *P12* shares a high nucleotide sequence identity (77.9%) with the cuticle-degrading protease encoding gene *spr1* of another nematode-trapping fungus *Monacrosporium megalosporum* [29].

**Figure 6:**
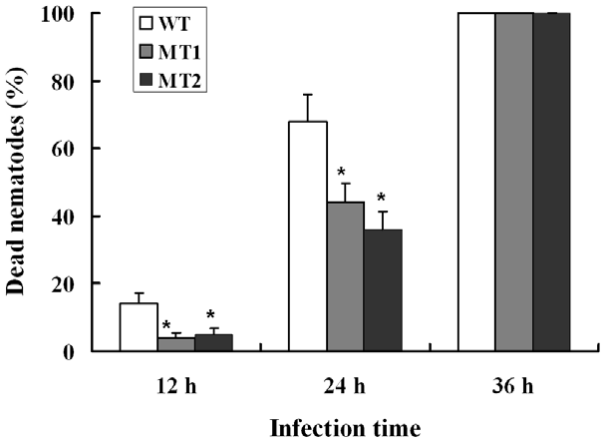
Disruption of the gene *p186* and nematocidal activity analysis. WT, wild-type strain. MT1 and MT2, mutant strains 1 and 2. Each point represents the average of five independent assays, and the bars represent the standard deviation values. *P<0.05 versus wild strain.

When *A. oligospora* was exposed to nematode extracts for 10 h, the transcription levels of *P12* and *P186* increased by 5.9- and 23.4-folds (Table S7) respectively, whereas those of *PII* and *P233* did not change significantly. In order to identify the role of *P186* in infection against nematodes, the gene *P186* was disrupted by homologous recombination according to the method described by Colot et al. [30]. The disruption removed 107 bp from the open reading frame of *P186* and was confirmed by PCR using genomic DNA as templates with primers (P186-5F and P186-3R). The phenotypic properties and nematocidal activities of the mutants were compared with the wild strains. No obvious differences in phenotypic properties, such as growth rates, spores number, traps number and morphology, were found between the mutants and the wild strain. However, disruption of *P186* greatly attenuated the pathogenicity of *A. oligospora*. The fatality rate of nematodes infected by P186-deletion mutants (*△P186*) decreased by 24–32% (Figure 6) at 24 h after infection. Our results suggested that *P186* likely play major roles during nematode infection by *A. oligospora*. In contrast, the lack of a significant change in *PII* expression level upon NE induction is consistent with an earlier finding that *PII* gene disruption had only a limited effect on the pathogenicity of *A. oligospora* [22].

Some fungi can produce secondary metabolites, including small molecular toxins that kill host cells before infection, generate a necrotrophic stage during infection, or disable host cellular functions after infection [31]. So far, 179 nematicidal compounds belonging to diverse chemical groups have been identified from nematophagous fungi, three (oligosporon, 4′,5′-dihydro-oligosporon and linoleic acid) of which were from *A. oligospora* [32]. Several enzyme families are commonly involved in the synthesis of these secondary metabolites in fungi, such as PKSs, NRPSs, and P450s. Genomic analysis identified five putative PKS genes and seven NRPS genes in the *A. oligospora* genome (Figure 5). Among the PKS genes, AOL_s00215g283 is likely involved in the production of 6-methyl salicylic acid (Figure S2). However, the functions of the seven NRPS genes could not be predicted based on the phylogenetic analysis (Figure S3). Of the P450 genes, our search against the fungal cytochrome P450 database (FCPD) [33] found that the 36 P450 (Table S8) genes in the *A. oligospora* genome could be classified into four classes (Group I P450, Group IV P450, pisatin demethylase-like P450, and CYP52 P450). P450 genes are known to be associated with the biosynthesis of diverse classes of secondary metabolites in other fungi [34].

### Proteomic and qPCR analyses of trap formation

Although the above-mentioned genes may play important roles in pathogenicity, the formation of trap likely involves other genes and is a prerequisite for infection, and serving as the indicator for lifestyle switch from saprophytic to predacious stages in nematode-trapping fungi [1], [2], [3], [4]. At present, little is known about the molecular mechanism of trap formation. To identify the proteins involved in trap formation, a proteomic study was performed and the profiles of intracellular proteins from *A. oligospora* cells at two developmental stages representing the early nematode extract (NE) induction stage (10 h after treatment with NE) and the late stage of trap formation (48 h after treatment with NE) (Figures S4 and 2B) were compared. As a negative control, mycelia incubated on medium without NE but with the solvent of NE (sterile deionized water) were analyzed. The expressions of 90 and 25 proteins were found up-regulated (*P*<0.05), while 16 and 94 were down-regulated (*P*<0.05) at 10 h and 48 h, respectively. Most of the proteins up-regulated at 10 h were involved in translation, posttranslational modification, amino acid metabolism, carbohydrate metabolism, energy conversion, cell wall and membrane biogenesis (Table S7). The results suggest very active growth and metabolism during the transition from vegetative hyphae to trap cells. In contrast, compared to those at the saprophytic stage, the expressions of most proteins up-regulated at 10 h were found either down-regulated or unchanged at 48 h when the traps were already formed, consistent with the hypothesis that the proteins up-regulated at 10 h were likely related to trap formation in *A. oligospora*.

Similar to other organisms, *A. oligospora* uses signaling cascades to alter its gene expression patterns in response to environmental changes. Genes encoding the components of common fungal signal transduction pathways are all found in the *A. oligospora* genome. Specifically, glycosylphosphatidylinositol-specific phospholipase C (AOL_s00109g54), mitogen-activated protein kinase (MAPK, AOL_s00173g235), serine/threonine protein phosphatase 2A (regulatory subunit, AOL_s00007g146), calcyclin binding protein (AOL_s00054g214), and Ca^2+^/calmodulin-dependent protein kinase (AOL_s00078g95) were all up-regulated (Table S7) during the formation of traps. This result is consistent with the importance of signal sensing and transduction in the shift from saprophytic to carnivorous lifestyles in *A. oligospora*.

Both hyphal growth and trap formation require energy. In *A. oligospora*, the tricarboxylic acid (TCA) cycle was up-regulated in response to NE, as indicated by the enhanced expression of genes in the TCA cycle, such as citrate synthase (AOL_s00079g361), aconitase (AOL_s00110g24), isocitrate dehydrogenase (AOL_s00075g141), succinyl-CoA synthetase (AOL_s00043g45), and malate dehydrogenase (AOL_s00210g140 and AOL_s00215g565) (Table S7). In addition, malate synthase (AOL_s00112g112) and isocitrate lyase (AOL_s00075g130), two key enzymes in the glyoxylate cycle, were up-regulated at 10 h (Table S7), indicating that this pathway is also important for trap formation. It has been reported that the glyoxylate cycle is associated with fungal virulence [35]. Taken together, our results suggest that the TCA cycle and the glyoxylate cycle are actively involved in *A. oligospora* trap formation, possibly by providing energy and substrates for macromolecule biosynthesis.

Cell division and cell cycle controls also play important roles during *A. oligospora* trap formation. In this study, the expression levels of Cdc37 (a molecular chaperone, AOL_s00043g594) and Mih1 (a phosphatases, AOL_s00176g31) (Table S7) were up-regulated during the formation of traps (10 h). In *S. cerevisiae* and *Schizosaccharomyces pombe*, Cdc37 is required for maintaining the protein level of a cyclin-dependent kinase Cdc28, a key enzyme for regulating the G1-S and the G2-M phase transitions [36]. Similarly, Mih1 promotes cell entry into mitosis by removing the inhibitory phosphorylation placed on Cdk1 by Wee1 in *S. cerevisiae* [37]. In addition, several cytoskeleton proteins such as two actin-binding proteins (AOL_s00097g552 and AOL_s00079g186), an actin-related protein (AOL_s00007g186), and a microtubule-binding protein (AOL_s00097g636) (Table S7) were significantly up-regulated at 10 h after exposure to NE. In summary, these proteins facilitate trap formation by increasing mitosis and cell proliferation.

The fungal cell wall is a complex structure composed of chitin, glucan and other polymers [38]. Traps are derived from specialized vegetative hyphae that have thicker and more robust cell walls than typical vegetative hyphae [39]. A large number of genes related to cell wall biosynthesis were identified in the *A. oligospora* genome. Proteomics analysis revealed that the expression levels of several proteins involved in cell wall synthesis, e.g. glycosyltransferase (AOL_s00097g268), glycosidase (AOL_s00083g375), phosphoglucomutase (AOL_s00054g87) (Table S7), were significantly up-regulated during trap formation (10 h). In addition, increased expressions at the mRNA level for 1,3-beta-glucan synthase (AOL_s00054g491), chitin synthases (AOL_s00210g37, AOL_s00075g119 and AOL_s00078g76) and glucosamine 6-phosphate synthetase (AOL_s00076g99) were also detected using qPCR (Table S7). Increased expression of those enzymes required for the biosynthesis of glucan, chitin and glycan undoubtedly facilitate new cell wall formation during trap formation.

Trap cells in nematode-trapping fungi contain numerous dense bodies that are related to peroxisomes [2], [5], [13]. A number of genes encoding peroxisomal biogenesis factors and related proteins were found (Table S9) in the *A. oligospora* genome. While no significant change in protein level was found in cells at 10 h after exposure to NE, the qPCR analyses of 10 selected peroxisomal genes revealed that four peroxisomal genes were significantly up-regulated during trap formation (Table S7). A previous study demonstrated that in *M. haptotylum*, the transcription level of a peroxisomal membrane protein (Peroxin-11) was up-regulated by 72% in nematode trapping knobs when compared to saprotrophic mycelia [13]. These data suggest that up-regulation of peroxisomal proteins is likely involved in the formation of dense bodies in trap cells in *A. oligospora*.

Glycogen is present in most fungi as a carbon storage molecule. The expression levels of glycogen phosphorylase (AOL_s00109g17) and hexokinase (AOL_s00112g89) were significantly up-regulated during *A. oligospora* trap formation (10 h) (Table S7), indicating accelerated glycogen degradation and glycolysis. In *M. haptotylum,* the glycogen phosphorylase (*gph1*) gene was also reported to be up-regulated during knob formation [13]. Moreover, the expression level of glycerol 3-phosphate dehydrogenase (AOL_s00054g748), a key enzyme in the synthesis of glycerol from dihydroxyacetone phosphate, increased by 5.7-fold during the formation of traps (Table S7). Accumulation of glycerol in response to NE was also confirmed experimentally (Figure S5). In contrast, degradation of fatty acids that generate other substrates for glycerol synthesis was down-regulated as indicated by the decreased expression of several beta-oxidation enzymes such as thiolase (AOL_s00210g122) and 3-hydroxyacyl-CoA dehydrogenase (AOL_s00110g113) (Table S7). Based on the proteomic and qPCR analyses, glycerol production during trap formation in *A. oligospora* likely occurred via the glycogen degradation and glycolysis pathways. In appressorium-forming fungi, such as *M. grisea*, glycerol plays a key role in generating hydrostatic turgor pressure to breach the host cuticles by a mechanical force [40]. The results here are consistent with the hypothesis that glycerol accumulation in *A. oligospora* likely function similarly to that of *M. grisea* during the penetration of nematode cuticle.

A previous study proposed that the capture of nematodes by *A. oligospora* was mediated by lectins located on the fungal cell surface [41]. An *A. oligospora* lectin (AOL) was subsequently identified [42]. However, AOL-deletion mutants showed similar saprophytic growth and nematode pathogenicity as their wild-type progenitor strain [42]. Our study revealed that seven genes encoding lectins with different sugar specificities were present in the *A. oligospora* genome (Table S10). Interestingly, none of the seven lectin genes, including AOL (AOL_s00080g288), showed any significant change in expression at either the mRNA or the protein level in response to NE.

The adhesive proteins comprising extracellular fibrils on trap cell surface can aid in nematode capturing [2], [5]. A total of 17 adhesion-associated protein-encoding genes (Table S10) were found in the *A. oligospora* genome. Our transcriptional analyses via qPCR revealed that the expressions of five adhesin encoding genes were up-regulated at 10 h (Table S7). One up-regulated adhesion gene, AOL_s00076g567, is a homolog of *MAD1*, an adhesin in the entomopathogenic fungus *Metarhizium anisopliae* that mediates its attachment to insects [43]. The other four adhesion proteins (AOL_s00043g50, AOL_s00007g5, AOL_s00210g231 and AOL_s00076g207) were up-regulated by 5.4-, 4.2-, 21.7- and 1.7-fold, respectively, during the formation of traps (10 h) (Table S7). These data support previous findings that adhesions on trap surfaces are important for capturing nematodes.

**Figure 7:**
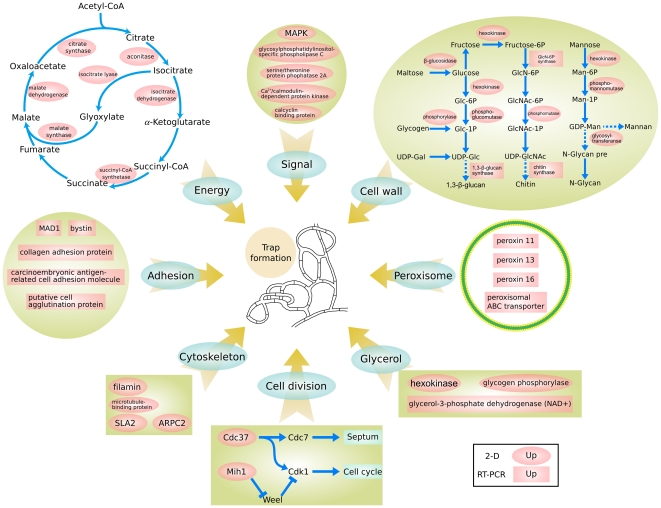
A proposed model for trap formation in *A. oligospora.* Protein information can be found in Table S7 . In this model, multiple fungal signal transduction pathways are activated by its nematode prey to further regulate downstream genes associated with diverse cellular processes such as energy metabolism, biosynthesis of the cell wall and adhesive proteins, cell division, glycerol accumulation and peroxisome biogenesis. Black letters in red background represented the up-regulated proteins. Oval represented the 2-D results and quadrate represented the qPCR results.

The combined genomic, proteomic, and qPCR data suggest a model of nematode trap formation in *A. oligospora* (Figure 7). In this model, *A. oligospora* recognizes nematode signals and activates a diversity of downstream cellular processes. These processes include (i) increased cell proliferation and septum formation with enhanced cell wall biosynthesis and cytoskeleton assembling; (ii) enhanced glycerol synthesis and accumulation leading to increased intracellular turgor pressure in preparation for host colonization and penetration within the nascent trapping cells; (iii) formation of dense bodies possibly involving the peroxisome biogenesis; and (iv) synthesis of adhesive proteins on the surface of the trapping cells to enhance nematode capture. These complex and coordinated processes are fueled by energy and building blocks supplied from the glyoxylate and TCA cycles.

## Discussion

Nematode trapping device is not only an important indicator of the switch from the saprophytic to the predacious lifestyle, but also a pivotal tool for capturing nematodes for nematode-trapping fungi [2], [4]. However, due to the lack of genome data of this group of fungi, there is currently limited information about the molecular mechanism of trap formation. Previous studies were mainly focused on the morphology and morphogenesis of traps. In this study, the major biological pathways, and the proteins and genes likely involved in trap formation in *A. oligospora* were first identified by proteomic and qPCR analyses. And a model on the formation of nematode trapping device in *A. oligospora* was proposed based on the combined genomic, proteomic, and qPCR data. However, the detailed spatial and temporal dynamics among the cellular processes, and their interactions during trap formation, remain to be elucidated.

*A. oligospora* is the first Orbiliomycete fungus to have its whole genome sequenced. Compared with other pathogenic and non-pathogenic fungi, *A. oligospora* contained abundant orphan genes not found in other sequenced fungi (53.64%) (Figure 3A), a result consistent with the phylogenomic analysis that *A. oligospora* is phylogenetically very distant from other sequenced Ascomycota (Figure 4). The *A. oligospora* specific genes may be related to its complex life-styles. Specifically, *A. oligospora* is not only a saprophyte, but also a nematode pathogen, a pathogen of other fungi, and a colonizer of plant roots [44]. The genes shared between *A. oligospora* and 10 other pathogenic and non-pathogenic ascomycete fungi (37.02%, Figure 3A) likely represent housekeeping genes for this group of organisms. Interestingly, *A. oligospora* share many more genes with pathogenic fungi than with non-pathogenic fungi. Those shared between *A. oligospora* and pathogens likely contribute to fungal pathogencity in general.

The distribution patterns of the pathogenicity-related gene families and putative PHI genes in different genome categories suggested that the ao-specific genes and the genes in the ao/pathogen category were more abundant than those in the ao/non-pathogen category. In fact, some pathogenicity-related gene families were completely absent in the ao/non-pathogen category. The genes specific to the ao/non-pathogen category are likely not involved in pathogenicity while the ao-specific genes may be very important for the pathogenicity of *A. oligospora*. Further analysis of PHI genes showed that carbohydrate-degrading enzymes (for example, polygalacturonase, xylanase and pectate lyase) represent a big proportion of ao-specific genes. Similarly, MFS transporter (11%), pectate lyase (6.8%) and short-chain dehydrogenase (6.8%) were the common genes specific to the ao/pathogens category.

Subtilisins and other pathogenicity-related genes play very important roles during infections [23], [34], [45]. As is typical for pathogens and saprobes of animals, the *A. oligospora* contains abundant pathogenicity genes encoding subtilisins and chitinases. Increasing evidences showed that increasing the copy number of virulence genes can improve the pathogenicity of pathogenic fungi. This strategy has been successfully used in *A. oligospora* [22] and several fungi used for biocontrol, such as *M. anisopliae* [46], *Beauveria bassiana* [47] and *Trichoderma harzianum* [48]. The qPCR analysis suggests that the proteases P12 and P186 likely play more important roles than PII during the infection process, and disruption of the gene *P186* further confirmed that P186 is an important pathogenicity factor in *A. oligospora*. Therefore, increasing the copy numbers of *P12* and *P186* through genetic engineering may effectively improve the pathogencity of *A. oligospora* and other fungi. Moreover, no obvious phenotypic differences were found between the mutants and the wild strains, suggesting that the *P186* may specifically work in pathogenicity without interfering with other metabolic and reproductive processes.

The nematode-trapping fungi can kill nematodes by producing secondary metabolites [32]. Several classes of secondary metabolite producing genes were found in the *A. oligospora* genome, suggesting that the potential repertoire of compounds is quite diverse as predicted. Some of these genes were not found in other closely related species (Figures S3 and S4) and could potentially contribute to producing novel metabolites. Further work may identify new nematicides from these secondary metabolite-producing genes in *A. oligospora*.

Attraction and recognition are the early steps during *A. oligospora* – nematodes interaction [49]. Nematodes are likely attracted by compounds released from the mycelia of nematode-trapping fungi [50]. However, the chemical attractant(s) involved in nematode-fungal recognition remain largely unknown. Recently, it was found that the nematoxic bacterium *Pseudomonas aeruginosa* could produce acylated homoserine lactones (AHL), the signal molecules in quorum sensing, as attractants for nematodes [51]. Interestingly, a homologous gene involved in biosynthesis of AHL was found in *A. oligospora* genome. Moreover, in our recent study, a “Trojan horse” mechanism of bacterial pathogenesis against nematodes was reported [52], in which the bacterium *Bacillus nematocida* B16 lures nematodes by emitting potent volatile organic compounds (VOCs), and seven VOCs are confirmed to lure nematodes, while the related genes had not been identified. These studies should help us identify the potential compounds involved in attraction and uncover the mechanism of recognition between the nematode-trapping fungi and nematodes.

Previous studies showed that lipid droplets accumulate in the trophic hyphae of *A. oligospora* at later stages of infection, and new vegetative mycelium developed from the trap that had originally captured the nematode when the lipid droplets disappeared [12]. Our results showed that several peroxisomal proteins as well as the key enzymes of the glyoxylate cycle were up-regulated during trap formation, which suggested that part of the nutrients released from the nematodes might first be converted to lipids by the fungus. The lipids were then degraded via the beta-oxidation pathway located on the peroxisome and further metabolized through the glyoxylate cycle to support growth of new vegetative hyphae. However, some of the enzymes involved in the beta-oxidation pathway were down-regulated during the early stage of trap formation. Further studies focusing on individual gene knockouts will be necessary to determine the roles of these enzymes in lipid metabolism during trap formation.

Fungi that are pathogenic to invertebrates, especially those targeting nematodes and insects, are of great importance for maintaining ecological balance in natural environments and for improving agricultural production [2], [53]. Here the first genome of this group of fungi was reported and the key features related to their pathogenicity were described. The information presented here on a nematophagous fungus should facilitate our understanding of these fascinating carnivorous fungi, and provide a basis to analyze the similarities and differences between nematophagous and other pathogenic fungi. These data also have important ramification for the practical development of improved biological control agents. Our proposed model for trap formation based on the comprehensive genomic, qPCR and proteomics analysis could serve as a roadmap for further investigating the molecule mechanism underlying the transition between saprophytic and predatory lifestyles in fungi.

## Methods

**Full methods** and any associated references are available in the Supporting information file (Text S1). The major methods described in the file were listed as follows. 1) Strain and growth conditions. 2) Genome sequencing, assembly, prediction and analysis. 3) Comparative genome, evolution and gene family analyses. 4) RIP analysis. 5) QPCR analysis. 6) Trap formation and fungus-nematode interaction. 7) Two-dimensional gel electrophoresis (2-DE) analysis. 8) Matrix assisted laser desorption ionization/time of flight (MALDI-TOF) analysis. 9) Disruption of the gene *p186.*

### Fungal strain

*A. oligospora* Fres. (ATCC24927) was purchased from the American Type Culture Collection (ATCC) and maintained on cornmeal agar (CMA). This fungus was originally isolated from soil in Sweden and provided to ATCC by Nordbring-Hertz B.

### Genome sequencing, assembly and analysis

The *A. oligospora* was sequenced by the ABI 3730 Sanger sequencing platform to a depth of 2 ×. An additional 34.6 × sequencing data coverage was provided by the Roche 454 Genome Sequencer Titanium/FLX platforms. GS *De Novo* Assembler developed by Roche was used to assemble the sequencing data. Repetitive sequences in the genome assembly were identified by searching the Repbase database [54] using RepeatMasker and by *de novo* repetitive sequence search using RepeatModeler (http://www.repeatmasker.org/RepeatModeler.html).

### Gene prediction and annotation

*Ab initio* gene prediction was performed on the genome assembly by Augustus, GlimmerHMM, and SNAP trained with transcript sequences of *A. oligospora* from this study, and by GeneMark-ES formulated for fungal genomes. A final set of gene models was selected by EvidenceModeler [55], combining *ab initio* gene predictions with supports by transcript alignments from *A. oligospora* and other fungal species. Predicted genes were annotated by BLAST searches against protein databases, and by InterProScan searches against protein domain databases.

### Non-coding RNAs analysis

tRNAs were predicted by tRNAscan-SE [56]. Ribosomal RNAs were identified by a BLAST search with known rRNA modules of other fungal genomes. Other non-coding RNAs, including snRNAs and miRNAs, were predicted by searching the Rfam database [57] using Infernal (http://infernal.janelia.org).

### Orthology and phylogenomic analysis

Predicted proteins in *A. oligospora* were compared with the predicted proteins of 10 sequenced fungal genomes. All proteins were searched against all other proteins in these genomes using BLASTP. The matches with E≤1e^−5^ and at least 30% sequence identity [58] over 60% of both protein lengths [15] were taken as homologous sequences. Bidirectional best hits (BBHs) [16] from the homologous sequences were taken as orthologous sequences. A total of 529 orthologous proteins were obtained and concatenated to infer the phylogenomic relationships among these taxa with PHYLIP [59] using different methods, including Neighbor-joining (NJ), Maximum parsimony (MP) and Maximum likelihood (ML).

### Multiple gene family analysis

Multigene families were constructed from the homologous sequences based on single linkage transitive closure [15]. A total of 2882 of the 11479 genes were clustering into 789 multigene families. Several gene families related to fungal pathogenicity were manually selected based on previous knowledge and gene annotations. The members of the pathogenicity-related gene families were expanded via homology search followed by clustering based on single linkage transitive closure. In addition, pathogenicity-related genes were also predicted by a whole genome blast analysis against the PHI gene database [21].

### Evolution analysis of pathogenicity-related gene families

The pathogenicity-related gene families of subtilisin, NRPS and PKS were selected to perform phylogenetic analysis. NJ tree was obtained using MEGA 4.1 [60] using bootstrap analysis with 1,000 replicates. MP tree was constructed using PAUP*4.0b8 [61] with a heuristic search (initial trees were obtained by 100 replicates with random addition and branch swapping with the TBR algorithm). Non-parameter bootstrap (1,000 replicates) was performed to assess the support level for each node on the MP trees. ML tree was obtained using PHYML version 3.0 [62]. Bootstrap analysis with 100 replicates was applied.

### Repeat-induced point mutation (RIP) analysis

The RIP indices, TpA/ApT and (CpA+TpG)/(ApC+GpT), were determined to detect RIP relics [20], [63], [64]. The AT content and RIP indices in all sequences were calculated. Windows of 500-bp and 200-bp with 100-bp shifts were performed separately, for the whole genome as well as individually for the coding regions, non-coding regions, exons, introns, multigene families, and repetitive sequences. RIP regions were detected in the 200-bp windows with 100-bp shifts with TpA/ApT ≥0.89 and (CpA+TpG)/(ApC+GpT) ≤1.03.

### Quantitative PCR

Quantitative PCR was conducted with 2 µl reverse transcribed product in a 7300 Real-Time PCR system (Applied Biosystems, California, USA) using Power SYBR Green PCR Master Mix (Applied Biosystems). 18S rDNA gene was used as the internal control. Fold changes were calculated using the formula 2^−(ΔΔCt)^, where ΔΔCt is ΔCt (treatment)-ΔCt (control), ΔCt is Ct (target gene) - Ct (18S), and Ct is the threshold cycle (User's Manual for ABI 7300 Real-Time PCR System).

### Proteomic analysis

Total proteins were extracted by following the method by Fernández-Acero et al. [65]. Approximately 1 mg of protein samples was brought to a final volume of 170 µl for 2-D analysis. Protein spots were manually excised from Coomassie Brilliant Blue G-250 (BBI) stained gels. Proteins were digested by trypsin for 16–20 h at 37°C. Peptides were analyzed by MALDI-TOF using a 4700 series Proteomics Analyzer (Applied Biosystems). Proteins were identified by searching PMF generated against a local database using Mascot algorithm of the GPS software.

### GenBank accession numbers

The Whole Genome Shotgun project has been deposited at DDBJ/EMBL/GenBank under the accession ADOT00000000. Other genes, accession nos. *PII*, X94121/AOL_s00076g4; *spr1*, AB120125; *Cdc37*, Z47813/AOL_s00043g594; *Mih1*, JO4846/AOL_s00176g31; *Cdc28*/*Cdk1*, Z36029; *Wee1*, X73966; Peroxin-11 (*PEX11*), EF419889; *gph1*, AY635194; *AOL*, X97093; *Mad1*, DQ438337/AOL_s00076g567.

## Supporting Information

Figure S1 Phylogenetic tree based on amino acid sequences of subtilases from *A. oligospora* and other fungi. The tree was constructed using three methods including ML, MP and NJ. The same topology was obtained by all the three methods. A. ML tree. B. MP tree. C. NJ tree. PTK: proteinase K-like, SF: subfamily, OSP: oxidatively stable proteases. Twenty four putative subtilase encoding genes were identified in the *A. oligospora* genome. Sequence alignment and phylogenetic analysis showed that these predicted subtilases in the *A. oligospora* genome can be grouped into four subtilisin families. Among the 24 putative subtilases, 20 genes were predicted to encode proteases in the proteinase K-like family and were further grouped into four subfamilies (SF2- SF5). Interestingly, SF5 contained 13 putative proteinase K-like subtilases, all of which were from *A. oligospora*, suggesting that this subfamily may be specific for *A. oligospora*. Gene AOL_s00076g4 corresponded to *PII*, a cuticle-degrading protease gene and a virulence factor in *A. oligospora*.
(JPG)

Figure S2 Phylogenetic tree based on amino acid sequences of PKS in *A. oligospora* and related fungi. Similar tree topology was obtained by all three analytical methods. A. ML tree. B. MP tree. C. NJ tree. Five putative PKS genes were identified in the *A. oligospora* genome. Phylogenetic analysis revealed that three of them (AOL_s00215g926, AOL_s00079g496, AOL_s00043g828) belonged to type I PKS, which were predicted to be involved in the biosynthesis of lovastatin. One gene (AOL_s00215g283) was grouped into type II PKS and it was clustered with the 6-methyl salicylic acid synthesis PKS genes. One gene (AOL_s00043g287) belonged to type III PKS and its function remains unidentified.
(JPG)

Figure S3 Phylogenetic tree based on amino acid sequences of NRPS in *A. oligospora* and related fungi. Similar tree topology was obtained by all three analytical methods. A. ML tree. B. MP tree. C. NJ tree. Seven putative NRPS genes were identified in the *A. oligospora* genome. One gene (AOL_s00215g415) is predicted to involve in the production of siderophore. AOL s00215g415 contained 11 introns and has a high molecular weight. Another gene (AOL_s00081g219) is orthologous to NRPS from *A. nidulans* and *A. fumigatus*. It contains 2 introns and its function remains unidentified. Three genes (AOL_s00210g71, AOL_s00188g306 and AOL_s00210g88) had no intron and they are orthologous to unidentified NRPS from *A. nidulans* and *A. fumigatus*. The remaining two genes (AOL_s00076g157 and AOL_s00188g124) belonged to NRPS-like enzymes, their functions are unknown.
(JPG)

Figure S4 Two-dimensional electrophoresis protein profiles of *A. oligospora*. Induced: hyphae treated with nematode extracts (NE) for 10 h, representing the initial stages of trap formation (A); and 48 h, representing the later stages of trap formation (B). Control: hyphae without NE treatment. Mycelia were collected from cultures without NE treatment (control), and treated with NE for 10 h and 48 h, respectively. Total proteins were extracted by using the protocol described previously. Proteins were separated by 2-DE as described in “supplemental materials and methods”, using IPG strips of pH 4–7. Differentially expressed proteins discussed in the text are labeled. 1. AOL_s00109g54; 2. AOL_s00007g146; 3. AOL_s00173g235; 4. AOL_s00054g214; 5. AOL_s00112g89; 6. AOL_s00004g627; 7. AOL_s00109g17; 8. AOL_s00110g144; 9. AOL_s00006g284; 10. AOL_s00078g394; 11. AOL_s00076g83; 12. AOL_s00083g229; 13. AOL_s00054g87; 14. AOL_s00004g628; 15. AOL_s00004g426; 16. AOL_s00054g899; 17. AOL_s00210g140; 18. AOL_s00112g112; 19. AOL_s00170g104; 20. AOL_s00043g45; 21. AOL_s00110g24; 22. AOL_s00075g130; 23. AOL_s00075g141; 24. AOL_s00004g494; 25. AOL_s00079g361; 26. AOL_s00215g818; 27. AOL_s00054g909; 28. AOL_s00004g362; 29. AOL_s00097g268; 30. AOL_s00083g375; 31. AOL_s00076g129; 32. AOL_s00176g31; 33. AOL_s00176g31.
(JPG)

Figure S5 The metabolite profile of the methanol extracts of culture broth and mycelia of *A. oligospora* cultivated on PDA by TLC. Lane 1, culture broth of *A. oligospora*. Lane 2, culture broth of *A. oligospora* treated with NE for 10 h. Lane 3, mycelia of *A. oligospora*. Lane 4, mycelia of *A. oligospora* treated with NE for 10 h. Black arrow indicated the white spot for glycerol.
(TIF)

Table S1 *A. oligospora* genomic data summary.
(DOC)

Table S2 Features of the *A. oligospora* genome.
(DOC)

Table S3 The numbers of bidirectional best hits (BBHs) identified between *A. oligospora* and other 10 fungal genomes. The total gene number of *A. oligospora* is 11479.
(DOC)

Table S4 Repetitive sequences in the *A. oligospora* genome.
(DOC)

Table S5 RIP analysis of *A. oligospora* genome. The AT content and RIP indices were calculated in all sequences, 500-bp windows and 200-bp windows with 100-bp shifts, separately, for the whole genome, coding and non-coding regions, exons, introns, multigene families and repetitive sequences. A positive response has been detected in the repetitive sequences. The RIP indices above the criteria are colored in red.
(DOC)

Table S6 Analysis of RIP regions of the *A. oligospora* genome. RIP regions were detected in the 200-bp windows with 100-bp shifts with TpA/ApT ≥0.89 and (CpA+TpG)/(ApC+GpT) ≤1.03. Almost one third of the genome sequences were covered with RIP-positive sequences.
(DOC)

Table S7 Differentially expressed genes in *A. oligospora* during the formation of traps (treated with NE for 10 h) in comparison to vegetative mycelia as revealed by proteomics or qPCR (in brackets) analysis. Proteins with changes greater than 1.5 folds between the two conditions are listed.
(DOC)

Table S8 List of cytochrome P450 from *A. oligospora*.
(DOC)

Table S9 Putative genes coding for peroxisomal proteins in the *A. oligospora* genome.
(DOC)

Table S10 Putative genes coding for lectins and adhesive proteins in the *A. oligospora* genome.
(DOC)

Text S1 Supporting text.
(DOC)
