# Supplementary figures and images for: Genomic and Proteomic Analyses of the Fungus Arthrobotrys oligospora Provide Insights into Nematode-Trap Formation
Source: PLoS Pathog. 2011 Sep 1;7(9):e1002179. doi: 10.1371/journal.ppat.1002179 (PMC3164635; doi:10.1371/journal.ppat.1002179)

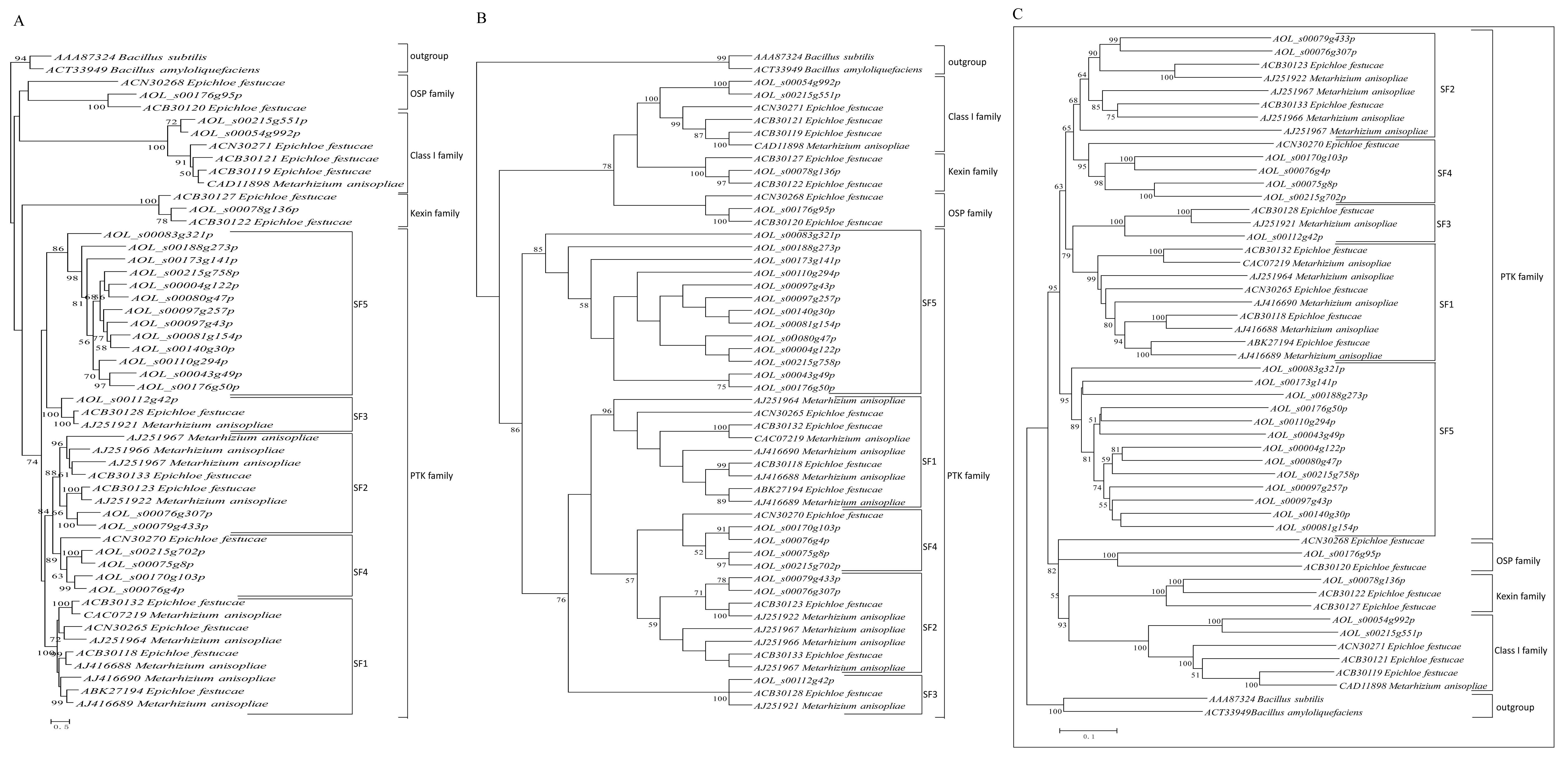

Supplement: Figure S1 — Phylogenetic tree based on amino acid sequences of subtilases from A. oligospora and other fungi. The tree was constructed using three methods including ML, MP and NJ. The same topology was obtained by all the three methods. A. ML tree. B. MP tree. C. NJ tree. PTK: proteinase K-like, SF: subfamily, OSP: oxidatively stable proteases. Twenty four putative subtilase encoding genes were identified in the A. oligospora genome. Sequence alignment and phylogenetic analysis showed that these predicted subtilases in the A. oligospora genome can be grouped into four subtilisin families. Among the 24 putative subtilases, 20 genes were predicted to encode proteases in the proteinase K-like family and were further grouped into four subfamilies (SF2- SF5). Interestingly, SF5 contained 13 putative proteinase K-like subtilases, all of which were from A. oligospora, suggesting that this subfamily may be specific for A. oligospora. Gene AOL_s00076g4 corresponded to PII, a cuticle-degrading protease gene and a virulence factor in A. oligospora. (JPG) [file ppat.1002179.s001.jpg]

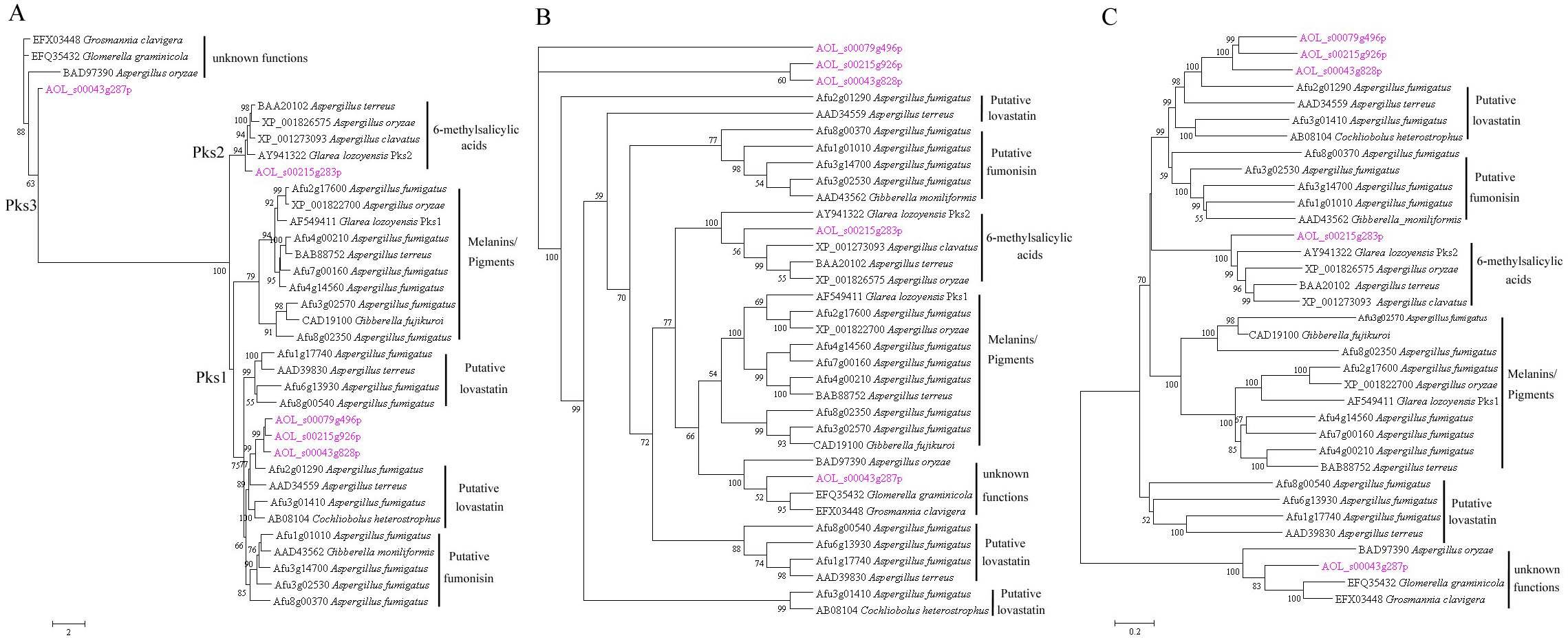

Supplement: Figure S2 — Phylogenetic tree based on amino acid sequences of PKS in A. oligospora and related fungi. Similar tree topology was obtained by all three analytical methods. A. ML tree. B. MP tree. C. NJ tree. Five putative PKS genes were identified in the A. oligospora genome. Phylogenetic analysis revealed that three of them (AOL_s00215g926, AOL_s00079g496, AOL_s00043g828) belonged to type I PKS, which were predicted to be involved in the biosynthesis of lovastatin. One gene (AOL_s00215g283) was grouped into type II PKS and it was clustered with the 6-methyl salicylic acid synthesis PKS genes. One gene (AOL_s00043g287) belonged to type III PKS and its function remains unidentified. (JPG) [file ppat.1002179.s002.jpg]

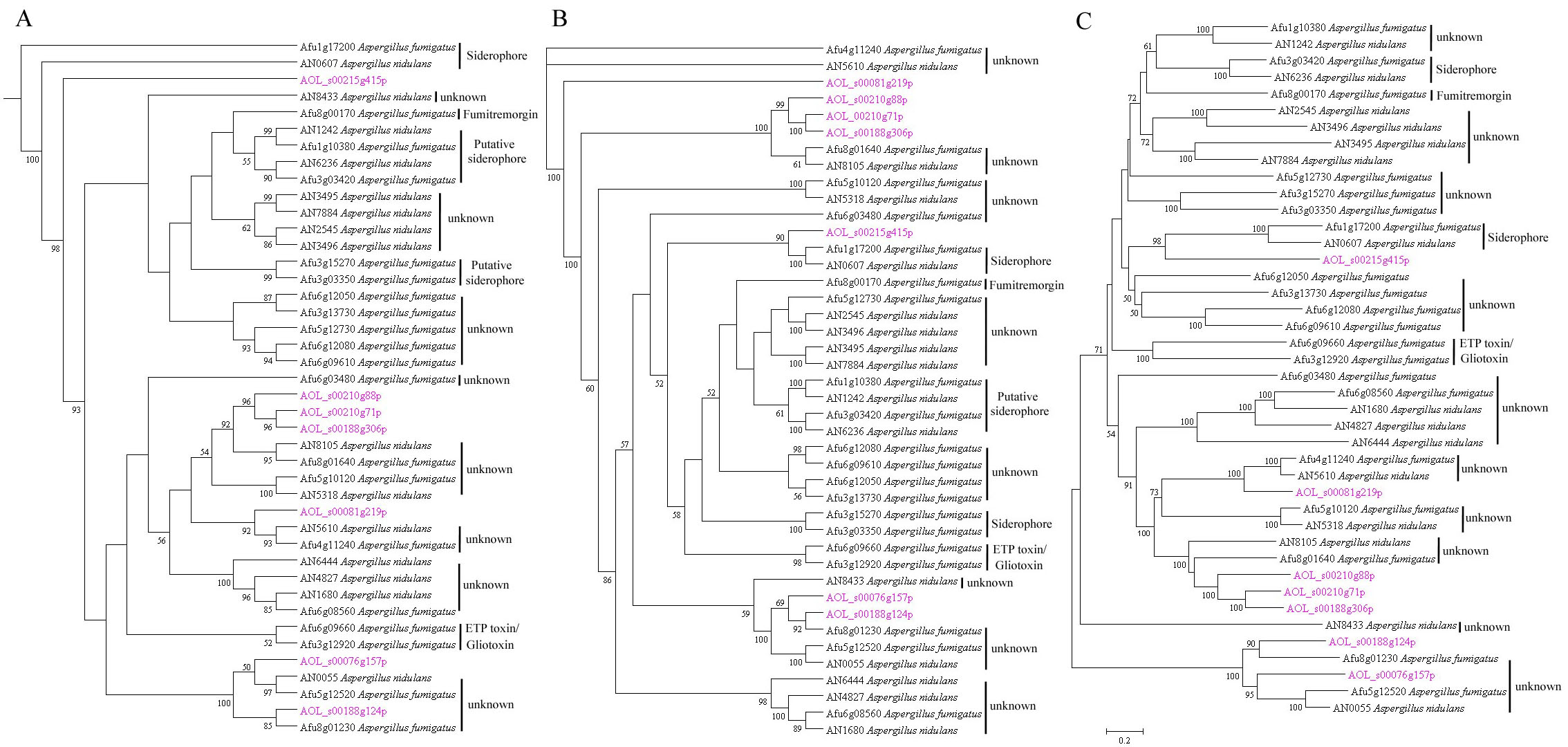

Supplement: Figure S3 — Phylogenetic tree based on amino acid sequences of NRPS in A. oligospora and related fungi. Similar tree topology was obtained by all three analytical methods. A. ML tree. B. MP tree. C. NJ tree. Seven putative NRPS genes were identified in the A. oligospora genome. One gene (AOL_s00215g415) is predicted to involve in the production of siderophore. AOL s00215g415 contained 11 introns and has a high molecular weight. Another gene (AOL_s00081g219) is orthologous to NRPS from A. nidulans and A. fumigatus. It contains 2 introns and its function remains unidentified. Three genes (AOL_s00210g71, AOL_s00188g306 and AOL_s00210g88) had no intron and they are orthologous to unidentified NRPS from A. nidulans and A. fumigatus. The remaining two genes (AOL_s00076g157 and AOL_s00188g124) belonged to NRPS-like enzymes, their functions are unknown. (JPG) [file ppat.1002179.s003.jpg]

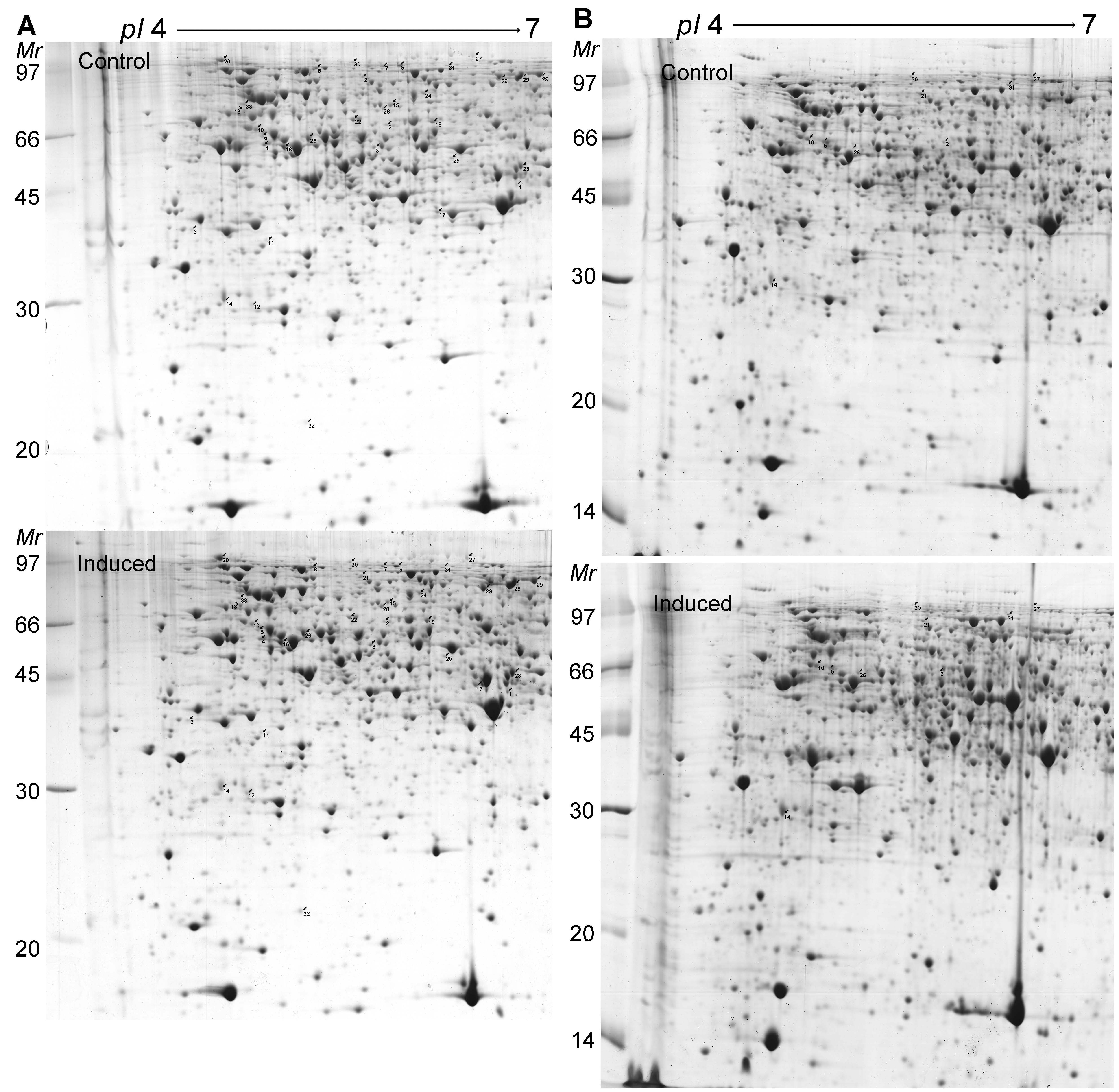

Supplement: Figure S4 — Two-dimensional electrophoresis protein profiles of A. oligospora. Induced: hyphae treated with nematode extracts (NE) for 10 h, representing the initial stages of trap formation (A); and 48 h, representing the later stages of trap formation (B). Control: hyphae without NE treatment. Mycelia were collected from cultures without NE treatment (control), and treated with NE for 10 h and 48 h, respectively. Total proteins were extracted by using the protocol described previously. Proteins were separated by 2-DE as described in “supplemental materials and methods”, using IPG strips of pH 4–7. Differentially expressed proteins discussed in the text are labeled. 1. AOL_s00109g54; 2. AOL_s00007g146; 3. AOL_s00173g235; 4. AOL_s00054g214; 5. AOL_s00112g89; 6. AOL_s00004g627; 7. AOL_s00109g17; 8. AOL_s00110g144; 9. AOL_s00006g284; 10. AOL_s00078g394; 11. AOL_s00076g83; 12. AOL_s00083g229; 13. AOL_s00054g87; 14. AOL_s00004g628; 15. AOL_s00004g426; 16. AOL_s00054g899; 17. AOL_s00210g140; 18. AOL_s00112g112; 19. AOL_s00170g104; 20. AOL_s00043g45; 21. AOL_s00110g24; 22. AOL_s00075g130; 23. AOL_s00075g141; 24. AOL_s00004g494; 25. AOL_s00079g361; 26. AOL_s00215g818; 27. AOL_s00054g909; 28. AOL_s00004g362; 29. AOL_s00097g268; 30. AOL_s00083g375; 31. AOL_s00076g129; 32. AOL_s00176g31; 33. AOL_s00176g31. (JPG) [file ppat.1002179.s004.jpg]

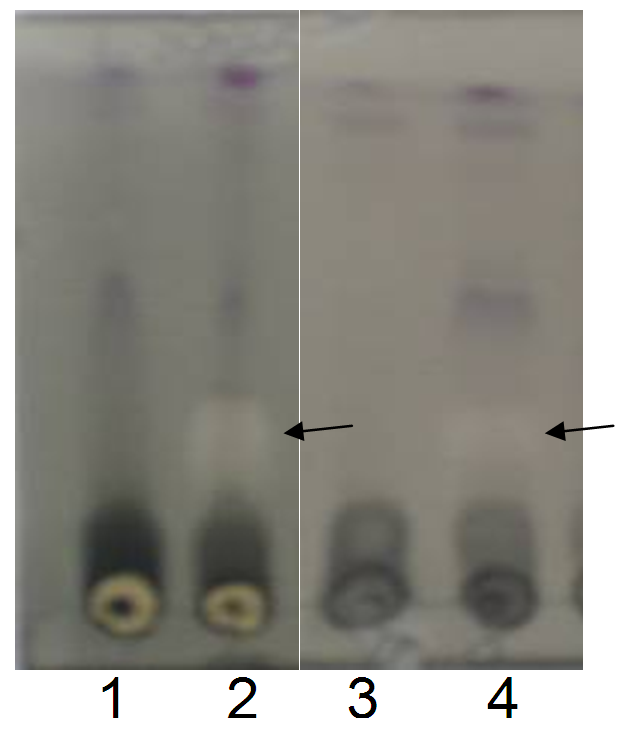

Supplement: Figure S5 — The metabolite profile of the methanol extracts of culture broth and mycelia of A. oligospora cultivated on PDA by TLC. Lane 1, culture broth of A. oligospora. Lane 2, culture broth of A. oligospora treated with NE for 10 h. Lane 3, mycelia of A. oligospora. Lane 4, mycelia of A. oligospora treated with NE for 10 h. Black arrow indicated the white spot for glycerol. (TIF) [file ppat.1002179.s005.tif]
