# Supplementary material for: Genomic and Proteomic Analyses of the Fungus Arthrobotrys oligospora Provide Insights into Nematode-Trap Formation
Source: PLoS Pathog. 2011 Sep 1;7(9):e1002179. doi: 10.1371/journal.ppat.1002179 (PMC3164635; doi:10.1371/journal.ppat.1002179)
Supplement: Table S1 — A. oligospora genomic data summary. (DOC) [file ppat.1002179.s006.doc]

**Table S1. *A. oligospora* genomic data summary.**

| Sequencing methods | Length (Mb) | Coverage (genome size set as 40 Mb) |
| --- | --- | --- |
| Sanger sequencing (plasmid library with 4-5 kb inserts) | 72 | 1.8 × |
| Sanger sequencing (fosmid library with 35 kb inserts) | 6 | 0.2 × |
| 454 pyrosequencing | 1384 | 34.6 × |
| Total | 1462 | 36.6 × |
