# Supplementary material for: Genomic and Proteomic Analyses of the Fungus Arthrobotrys oligospora Provide Insights into Nematode-Trap Formation
Source: PLoS Pathog. 2011 Sep 1;7(9):e1002179. doi: 10.1371/journal.ppat.1002179 (PMC3164635; doi:10.1371/journal.ppat.1002179)
Supplement: Table S2 — Features of the A. oligospora genome. (DOC) [file ppat.1002179.s007.doc]

**Table S2. Features of the *A. oligospora*** genome.

| General features | Value |
| --- | --- |
| Size of assembled genome including gaps (bp) | 40,072,829 |
| Number of scaffolds larger than 2 kb | 215 |
| Number of scaffolds | 323 |
| Length of gaps within scaffolds (bp) | 106,613 |
| N50 scaffold size (bp) | 2,037,373 |
| N50 contig size (bp) | 575,766 |
| GC content (%) | 44.45 |
| Repeat regions (%) | 0.47 |
| Coding regions (%) | 48.47 |
| Number of predicted gene models | 11,479 |
| Number of single-exon genes | 2616 |
| Average gene length (bp) | 1690 |
| Average number of introns per multi-exon gene | 2.8 |
| Average intron size (bp) | 89 |
| Average exon size (bp) | 473 |
| Number of tRNA genes | 145 |
