# Supplementary material for: Genomic and Proteomic Analyses of the Fungus Arthrobotrys oligospora Provide Insights into Nematode-Trap Formation
Source: PLoS Pathog. 2011 Sep 1;7(9):e1002179. doi: 10.1371/journal.ppat.1002179 (PMC3164635; doi:10.1371/journal.ppat.1002179)
Supplement: Table S3 — The numbers of bidirectional best hits (BBHs) identified between A. oligospora and other 10 fungal genomes. The total gene number of A. oligospora is 11479. (DOC) [file ppat.1002179.s008.doc]

**Table S3**. The numbers of bidirectional best hits (BBHs) identified between *A. oligospora* and other 10 fungal genomes. The total gene number of *A. oligospora* is 11479.

| Fungi | Total gene numbers | Number of BBHs |
| --- | --- | --- |
| *Aspergillus fumigatus* | 9887 | 3910 |
| *Chaetomium globosum* | 11048 | 3210 |
| *Coccidioides immitis* | 10440 | 3500 |
| *Aspergillus nidulans* (syn. *Emericella nidulans*) | 9410 | 3439 |
| *Fusarium graminearum* | 13321 | 4101 |
| *Histoplasma capsulatum* | 9251 | 3100 |
| *Magnaporthe grisea* | 12832 | 3690 |
| *Neurospora crassa* | 9841 | 3540 |
| *Saccharomyces cerevisiae* | 5902 | 1868 |
| *Verticillium dahliae* | 10535 | 3772 |
