# Supplementary material for: Genomic and Proteomic Analyses of the Fungus Arthrobotrys oligospora Provide Insights into Nematode-Trap Formation
Source: PLoS Pathog. 2011 Sep 1;7(9):e1002179. doi: 10.1371/journal.ppat.1002179 (PMC3164635; doi:10.1371/journal.ppat.1002179)
Supplement: Table S4 — Repetitive sequences in the A. oligospora genome. (DOC) [file ppat.1002179.s009.doc]

**Table S4.** **Repetitive sequences in the *A. oligospora* genome.**

| Types of repetitive sequences | Number | Length (bp) |
| --- | --- | --- |
| SINEs | 26 | 1,967 |
| LINEs | 502 | 40,062 |
| LTR elements | 891 | 80,667 |
| DNA elements | 1076 | 67,748 |
| Unclassified | 47 | 5,968 |
