# Supplementary material for: Genomic and Proteomic Analyses of the Fungus Arthrobotrys oligospora Provide Insights into Nematode-Trap Formation
Source: PLoS Pathog. 2011 Sep 1;7(9):e1002179. doi: 10.1371/journal.ppat.1002179 (PMC3164635; doi:10.1371/journal.ppat.1002179)
Supplement: Table S5 — RIP analysis of A. oligospora genome. The AT content and RIP indices were calculated in all sequences, 500-bp windows and 200-bp windows with 100-bp shifts, separately, for the whole genome, coding and non-coding regions, exons, introns, multigene families and repetitive sequences. A positive response has been detected in the repetitive sequences. The RIP indices above the criteria are colored in red. (DOC) [file ppat.1002179.s010.doc]

**Table S5**. RIP analysis of *A. oligospora* genome. The AT content and RIP indices were calculated in all sequences, 500-bp windows and 200-bp windows with 100-bp shifts, separately, for the whole genome, coding and non-coding regions, exons, introns, multigene families and repetitive sequences, etc. A positive response has been detected in the repetitive sequences with TpA/ApT ≥ 0.89 and (CpA+TpG)/(ApC+GpT) ≤ 1.03. The RIP indices above the criteria are colored in red.

| Sequence categories | Windows | Number | Length | AT content | TpA/ApT | (CpA+TpG)/(ApC+GpT) |
| --- | --- | --- | --- | --- | --- | --- |
| Coding regions | Total |  |  | 0.52 | 0.75 | 1.17 |
|  | 200-100bp | 176997 | 200.00±0.00 | 0.53±0.05 | 0.79±0.36 | 1.20±0.30 |
|  | 500bp | 33013 | 500.00±0.00 | 0.53±0.03 | 0.76±0.26 | 1.18±0.19 |
|  | All sequences | 11479 | 1692.12±1260.32 | 0.52±0.03 | 0.76±0.13 | 1.17±0.14 |
| Exons | Total |  |  | 0.51 | 0.70 | 1.19 |
|  | 200-100bp | 125731 | 200.00±0.00 | 0.51±0.04 | 0.75±0.40 | 1.22±0.31 |
|  | 500bp | 20075 | 500.00±0.00 | 0.51±0.03 | 0.72±0.21 | 1.20±0.20 |
|  | All sequences | 36375 | 472.94±601.89 | 0.52±0.05 | 0.71±0.35 | 1.28±0.48 |
| Coding regions of multigene families | Total |  |  | 0.53 | 0.77 | 1.16 |
|  | 200-100bp | 46274 | 200.00±0.00 | 0.53±0.04 | 0.79±0.36 | 1.20±0.29 |
|  | 500bp | 8675 | 500.00±0.00 | 0.53±0.03 | 0.78±0.31 | 1.18±0.18 |
|  | All sequences | 2882 | 1756.09±1069.99 | 0.53±0.02 | 0.77±0.11 | 1.17±0.12 |
| Exons of multigene families | Total |  |  | 0.52 | 0.70 | 1.19 |
|  | 200-100bp | 30227 | 200.00±0.00 | 0.52±0.04 | 0.75±0.41 | 1.22±0.29 |
|  | 500bp | 4607 | 500.00±0.00 | 0.52±0.03 | 0.72±0.18 | 1.20±0.18 |
|  | All sequences | 10872 | 401.99±503.28 | 0.52±0.05 | 0.71±0.39 | 1.29±0.48 |
| Introns of multigene families | Total |  |  | 0.61 | 1.06 | 0.98 |
|  | 200-100bp | 506 | 200.00±0.00 | 0.57±0.06 | 0.86±0.27 | 1.19±0.28 |
|  | 500bp | 43 | 500.00±0.00 | 0.57±0.05 | 0.85±0.17 | 1.14±0.15 |
|  | All sequences | 7990 | 86.44±67.35 | 0.62±0.06 | 1.20±0.57 | 1.01±0.40 |
| Whole genome | Total |  |  | 0.56 | 0.83 | 1.15 |
|  | 200-100bp | 400406 | 200.00±0.00 | 0.55±0.07 | 0.84±0.35 | 1.19±0.32 |
|  | 500bp | 80051 | 500.00±0.00 | 0.55±0.06 | 0.82±0.21 | 1.16±0.20 |
|  | All sequences | 215 | 186385.25±572813.80 | 0.79±0.12 | 1.00±0.10 | 0.94±0.15 |
| Introns | Total |  |  | 0.61 | 1.05 | 0.98 |
|  | 200-100bp | 2158 | 200.00±0.00 | 0.57±0.06 | 0.85±0.29 | 1.22±0.35 |
|  | 500bp | 214 | 500.00±0.00 | 0.56±0.04 | 0.84±0.19 | 1.19±0.23 |
|  | All sequences | 24896 | 89.19±86.47 | 0.62±0.06 | 1.20±0.58 | 1.01±0.39 |
| Noncoding regions | Total |  |  | 0.58 | 0.88 | 1.14 |
|  | 200-100bp | 189037 | 200.00±0.00 | 0.58±0.09 | 0.89±0.34 | 1.17±0.33 |
|  | 500bp | 35451 | 500.00±0.00 | 0.58±0.08 | 0.88±0.20 | 1.15±0.22 |
|  | All sequences | 11694 | 1765.78±2318.40 | 0.59±0.05 | 0.89±0.16 | 1.14±0.17 |
| Repetitive sequences | Total |  |  | 0.73 | 0.99 | 1.05 |
|  | 200-100bp | 5748 | 200.00±0.00 | 0.78±0.11 | 1.04±0.67 | 1.00±0.37 |
|  | 500bp | 1034 | 500.00±0.00 | 0.78±0.10 | 1.01±0.13 | 0.97±0.21 |
|  | All sequences | 3439 | 248.66±820.76 | 0.60±0.13 | 1.17±2.06 | 1.49±1.54 |
