# Supplementary material for: Genomic and Proteomic Analyses of the Fungus Arthrobotrys oligospora Provide Insights into Nematode-Trap Formation
Source: PLoS Pathog. 2011 Sep 1;7(9):e1002179. doi: 10.1371/journal.ppat.1002179 (PMC3164635; doi:10.1371/journal.ppat.1002179)
Supplement: Table S6 — Analysis of RIP regions of the A. oligospora genome. RIP regions were detected in the 200-bp windows with 100-bp shifts with TpA/ApT ≥0.89 and (CpA+TpG)/(ApC+GpT) ≤1.03. Almost one third of the genome sequences were covered with RIP-positive sequences. (DOC) [file ppat.1002179.s011.doc]

**Table S6**. RIP regions analysis of *A. oligospora* genome. RIP regions were detected in the 200-bp windows with 100-bp shifts with TpA/ApT ≥ 0.89 and (CpA+TpG)/(ApC+GpT) ≤ 1.03. Almost one third of the genome sequences were covered with RIP-positive sequences.

| Sequence categories | Total number | RIP regions identified | Percent (%) | Total length | RIP regions coverage | Percent (%) |
| --- | --- | --- | --- | --- | --- | --- |
| Coding regions | 11479 | 8845 | 77.05 | 19423789 | 4955000 | 25.51 |
| Exons | 36375 | 8384 | 23.05 | 17203295 | 2925700 | 17.01 |
| Coding regions in multigene families | 2882 | 2348 | 81.47 | 5061048 | 1333300 | 26.34 |
| Exons in multigene families | 10872 | 2109 | 19.40 | 4370397 | 693500 | 15.87 |
| Introns in multigene families | 7990 | 72 | 0.90 | 690651 | 19000 | 2.75 |
| Whole genome | 215 | 215 | 100.00 | 39966211 | 13714700 | 34.32 |
| Intron | 24896 | 284 | 1.14 | 2220494 | 83500 | 3.76 |
| Noncoding regions | 11694 | 9093 | 77.76 | 20542422 | 7851000 | 38.22 |
| Repetitive sequences | 3439 | 416 | 12.10 | 855058 | 439000 | 51.34 |
