# Supplementary material for: Genomic and Proteomic Analyses of the Fungus Arthrobotrys oligospora Provide Insights into Nematode-Trap Formation
Source: PLoS Pathog. 2011 Sep 1;7(9):e1002179. doi: 10.1371/journal.ppat.1002179 (PMC3164635; doi:10.1371/journal.ppat.1002179)
Supplement: Table S7 — Differentially expressed genes in A. oligospora during the formation of traps (treated with NE for 10 h) in comparison to vegetative mycelia as revealed by proteomics or qPCR (in brackets) analysis. Proteins with changes greater than 1.5 folds between the two conditions are listed. (DOC) [file ppat.1002179.s012.doc]

**Table** **S7. Differentially expressed genes in *A. oligospora* during the formation of traps (treated with NE for 10 h) in comparison to vegetative mycelia as revealed by proteomics or qPCR (in brackets) analysis.** Proteins with changes greater than 1.5 folds between the two conditions are listed.

| Gene ID | KOG/COG description | 10 hr | | 48 hr | |
| --- | --- | --- | --- | --- | --- |
|  |  | Fold | t test | Fold | t test |
| Signal transduction mechanisms | | | | | |
| AOL_s00109g54 | Glycosylphosphatidylinositol-specific phospholipase C | +1.78 | 0.05 | nd | / |
| AOL_s00007g146 | Serine/threonine protein phosphatase 2A, regulatory subunit | +2.46 | 0 | -2.82 | 0 |
| AOL_s00173g235 | Mitogen-activated protein kinase | +1.91 | 0.02 | nd | / |
| AOL_s00078g95 | Ca2+/calmodulin-dependent protein kinase | +2.21 | 0.02 | nd | / |
| AOL_s00054g214 | Calcyclin binding protein | +2.07 | 0.01 | nd | / |
| Translation, ribosomal structure and biogenesis | | | | | |
| AOL_s00054g867 | Translation initiation factor eIF3, p35 subunit | +2.28 | 0.01 | nd | / |
| AOL_s00043g682 | Translation initiation factor 3, subunit d (eIF-3d) | +2.19 | 0.01 | -2.93 | 0 |
| AOL_s00081g271 | Phenylalanyl-tRNA synthetase | +2.11 | 0 | nd | / |
| AOL_s00054g814 | Translation initiation factor 3, subunit b (eIF-3b) | +1.85 | 0 | nd | / |
| AOL_s00173g186 | Cytoplasmic tryptophanyl-tRNA synthetase | +2.78 | 0 | nd | / |
| AOL_s00173g324 | Elongation factor 1 beta/delta chain | +3.41 | 0.02 | nd | / |
| AOL_s00078g222 | Nuclear localization sequence binding protein | +1.95 | 0.05 | nd | / |
| AOL_s00112g117 | RNA polymerase II general transcription factor BTF3 and related proteins | +4.09 | 0.05 | nd | / |
| Posttranslational modification, protein turnover, chaperones | | | | | |
| AOL_s00078g242 | Chaperonin complex component, TCP-1 delta subunit (CCT4) | +2.32 | 0.03 | nd | / |
| AOL_s00004g635 | Ubiquitin-specific protease | +1.77 | 0.04 | nd | / |
| AOL_s00004g618 | Ubiquitin activating enzyme UBA1 | +2.30 | 0 | nd | / |
| AOL_s00193g46 | Multifunctional chaperone (14-3-3 family) | +2.50 | 0 | nd | / |
| AOL_s00112g87 | Ubiquitin-specific protease UBP14 | +2.03 | 0 | nd | / |
| AOL_s00215g489 | Predicted ubiquitin regulatory protein | +2.36 | 0.01 | nd | / |
| AOL_s00210g337 | Heat shock 70kDa protein 5 | +1.82 | 0.04 | nd | / |
| AOL_s00110g132 | Alkyl hydroperoxide reductase/peroxiredoxin | +1.66 | 0.04 | nd | / |
| AOL_s00076g65 | Molecular chaperone (small heat-shock protein Hsp26/Hsp42) | +2.33 | 0 | nd | / |
| AOL_s00097g49 | COP9 signalosome, subunit CSN1 | +1.96 | 0.02 | nd | / |
| AOL_s00079g328 | SCF ubiquitin ligase, Skp1 component | -2.25 | 0 | nd | / |
| AOL_s00076g498 | Molecular chaperone | -9.26 | 0.00 | nd | / |
| Replication, recombination and repair | | | | | |
| AOL_s00215g413 | DNA damage inducible protein | +1.93 | 0.03 | nd | / |
| AOL_s00079g321 | Nucleotide excision repair factor NEF2, RAD23 component | +1.86 | 0 | nd | / |
| RNA processing and modification | | | | | |
| AOL_s00004g556 | dsRNA-specific nuclease Dicer and related ribonucleases | -3.03 | 0.05 | nd | / |
| Nucleotide transport and metabolism | | | | | |
| AOL_s00079g65 | Adenylosuccinate synthase | +2.64 | 0.02 | nd | / |
| AOL_s00081g99 | Ribose-phosphate pyrophosphokinase | +1.99 | 0.01 | nc | / |
| AOL_s00215g155 | 3'(2'), 5'-Bisphosphate nucleotidase | +1.67 | 0.04 | nd | / |
| Amino acid transport and metabolism | | | | | |
| AOL_s00076g583 | Branched chain aminotransferase | +2.49 | 0.01 | uc | / |
| AOL_s00215g888 | Cysteine desulfurase (NFS1) | +4.87 | 0 | nd | / |
| AOL_s00043g753 | Aromatic-L-amino-acid decarboxylase | +1.75 | 0.04 | -2.60 | 0 |
| AOL_s00080g371 | Glutamate dehydrogenase | +3.05 | 0 | -8.33 | 0 |
| AOL_s00173g271 | Gamma-glutamyltransferase | +1.66 | 0.03 | nd | / |
| AOL_s00054g637 | Glycine hydroxymethyltransferase | +3.0 | 0.01 | nd | / |
| AOL_s00054g941 | Isocitrate dehydrogenase (NAD+) | -8.65 | 0.00 | ns | / |
| Carbohydrate transport and metabolism | | | | | |
| AOL_s00112g89 | Hexokinase | +1.82 | 0 | +2.07 | 0.05 |
| AOL_s00004g627 | Ribokinase | +1.94 | 0 | nd | / |
| AOL_s00109g17 | Glycogen phosphorylase | +3.95 | 0 | nd | / |
| AOL_s00110g144 | alpha,alpha-Trehalose-phosphate synthase | +2.77 | 0 | nd | / |
| AOL_s00006g284 | alpha-Glucosidase | +2.71 | 0 | nd | / |
| AOL_s00078g394 | Transaldolase | +1.77 | 0 | +2.93 | 0 |
| AOL_s00076g83 | Predicted phosphoglycerate mutase | +1.86 | 0.03 | nd | / |
| AOL_s00083g229 | 6-Phosphogluconolactonase | +1.65 | 0.05 | nd | / |
| AOL_s00054g87 | Phosphoglucomutase/phosphomannomutase | +1.75 | 0 | nd | / |
| AOL_s00004g628 | Oxalate decarboxylase | +2.06 | 0 | +3.44 | 0.02 |
| AOL_s00054g465 | Phosphoglucomutase/phosphomannomutase | (-2.65) | (0.01) | nd | / |
| AOL_s00054g465 | Phosphoglucomutase/phosphomannomutase | (-3.21) | (0.02) | nd | / |
| AOL_s00004g426 | Dihydroxyacetone kinase/glycerone kinase | -1.82 | 0.03 | nd | / |
| AOL_s00054g899 | Enolase | -1.71 | 0.01 | nd | / |
| Lipid transport and metabolism | | | | | |
| AOL_s00004g288 | 3-oxoacyl-[acyl-carrier protein] reductase | +3.28 | 0 | nd | / |
| AOL_s00081g51 | Phosphatidylinositol transfer protein SEC14 and related proteins | +1.70 | 0.01 | nd | / |
| AOL_s00043g424 | Leukotriene A4 hydrolase | +1.85 | 0.03 | nd | / |
| AOL_s00210g122 | 3-oxoacyl CoA thiolase | -2.27 | 0 | nd | / |
| AOL_s00110g113 | 3-hydroxyacyl-CoA dehydrogenase | -2.04 | 0.02 | nd | / |
| Energy production and conversion | | | | | |
| AOL_s00210g140 | NAD-dependent malate dehydrogenase | +2.99 | 0 | nd | / |
| AOL_s00112g112 | Malate synthase | 2.38 | 0.05 | ns | / |
| AOL_s00170g104 | Voltage-gated shaker-like K+ channel, subunit beta/KCNAB | +1.66 | 0.01 | ns | / |
| AOL_s00043g45 | Succinyl-CoA synthetase, beta subunit | +1.94 | 0.02 | nd | / |
| AOL_s00215g565 | NAD-dependent malate dehydrogenase | (+1.57) | (0.03) | nd | / |
| AOL_s00110g24 | Aconitase/homoaconitase (aconitase superfamily) | +1.90 | 0.01 | -2.11 | 0.04 |
| AOL_s00054g748 | Glycerol-3-phosphate dehydrogenase | (+5.66) | (0.05) | nd | / |
| AOL_s00075g130 | Isocitrate lyase | +2.12 | 0 | nd | / |
| AOL_s00075g141 | NADP-dependent isocitrate dehydrogenase | +1.95 | 0 | nd | / |
| AOL_s00004g494 | 5'-AMP-activated protein kinase, gamma subunit | +1.92 | 0.02 | nd | / |
| AOL_s00079g361 | Citrate synthase | +1.86 | 0.02 | nd | / |
| AOL_s00215g818 | Aldehyde dehydrogenase | +2.16 | 0 | +4.59 | 0 |
| AOL_s00054g909 | Pyruvate carboxylase | +7.46 | 0.01 | -2.66 | 0.05 |
| AOL_s00007g287 | Function unknown | +2.59 | 0 | -2.57 | 0.02 |
| AOL_s00004g362 | Phosphoenolpyruvate carboxykinase (ATP) | +1.98 | 0 | nd | / |
| AOL_s00007g381 | Cytochrome c oxidase, subunit Va/COX6 | +4.41 | 0 | nd | / |
| AOL_s00097g316 | Mitochondrial phosphate carrier protein | +2.48 | 0 | nd | / |
| AOL_s00006g249 | Mitochondrial ADP/ATP carrier proteins | -3.28 | 0.01 | nd | / |
| Cell wall/membrane/envelope biogenesis | | | | | |
| AOL_s00097g268 | Glycosyltransferase | +2.63 | 0 | nd | / |
| AOL_s00083g375 | Beta-glucosidase-related glycosidases | +1.82 | 0 | -3.64 | 0.01 |
| AOL_s00076g129 | Rhs family protein | +3.72 | 0 | +8.32 | 0 |
| AOL_s00075g119 | Chitin synthase/hyaluronan synthase (glycosyltransferases) | (+1.80) | (0.03) | nd | / |
| AOL_s00054g491 | 1,3-beta-Glucan synthase/callose synthase catalytic subunit | (+1.87) | (0.04) | nd | / |
| AOL_s00210g37 | Chitin synthase/hyaluronan synthase (glycosyltransferases) | (+2.31) | (0)) | nd | / |
| AOL_s00076g99 | Glucosamine 6-phosphate synthetases | (+4.49) | (0.01) | nd | / |
| AOL_s00078g76 | Chitin synthase/hyaluronan synthase (glycosyltransferases) | (+2.36) | (0.03) | nd | / |
| Cytoskeleton | | | | | |
| AOL_s00097g552 | Actin-binding cytoskeleton protein (filamin) | +1.67 | 0.01 | nd | / |
| AOL_s00007g186 | Actin-related protein Arp2/3 complex (ARPC2) | +3.82 | 0.02 | -3.66 | 0 |
| AOL_s00007g138 | Drebrins and related actin binding proteins | +2.71 | 0 | nd | / |
| AOL_s00079g186 | Actin-binding protein (SLA2) | +4.24 | 0 | nd | / |
| AOL_s00097g636 | Microtubule-binding protein | +1.94 | 0.01 | nd | / |
| Cell cycle control, cell division | | | | | |
| AOL_s00176g31 | M-phase inducer phosphatase (MIH1) | +3.35 | 0.03 | nd | / |
| AOL_s00043g594 | Cell division cycle 37 protein (CDC37) | +3.75 | 0 | nd | / |
| AOL_s00054g531 | Metacaspase involved in regulation of apoptosis | -1.89 | 0.05 | ns | / |
| Adhesion proteins | | | | | |
| AOL_s00043g50 | Cell adhesion complex protein (bystin) | (+5.43) | (0.01) | nd | / |
| AOL_s00076g207 | Collagen adhesion protein | (+1.72) | 0.03 | nd | / |
| AOL_s00210g231 | Carcinoembryonic antigen-related cell adhesion molecule | (+21.72) | (0.02) | nd | / |
| AOL_s00007g5 | Putative cell agglutination protein | (+4.23) | (0.04) | nd | / |
| AOL_s00076g567 | Adhesin protein (Mad1) | (+1.71) | (0.05) | nd | / |
| Peroxisome | | | | | |
| AOL_s00083g431 | Peroxisomal biogenesis protein (peroxin 11B) | (+2.67) | (0.04) | nd |  |
| AOL_s00007g540 | Peroxisomal biogenesis protein (peroxin 16) | (+1.78) | (0.05) | nd | / |
| AOL_s00004g606 | Peroxisomal ABC transporter (PXA1) | (+1.92) | (0.05) | nd | / |
| AOL_s00054g525 | Peroxisomal biogenesis protein (peroxin 13) | (+1.84) | (0.05) | nd | / |
| Proteases | | | | | |
| AOL_s00170g103 | Serine protease [*Monacrosporium megalosporum*] | (+5.89) | (0.03) | nd | / |
| AOL_s00215g702 | Cuticle-degrading serine protease | (+23.36) | (0) | nd | / |
| AOL_s00112g42 | Subtilisin-related protease | -2.70 | 0.03 | nd | / |
| Coenzyme transport and metabolism | | | | | |
| AOL_s00083g125 | Phosphoglycerate dehydrogenase and related dehydrogenases | +1.88 | 0 | nd |  |
| Secondary metabolites biosynthesis, transport and catabolism | | | | | |
| AOL_s00081g10 | Alcohol dehydrogenase, class V | +2.45 | 0.01 | +3.10 | 0.02 |
| AOL_s00193g126 | Sorbitol dehydrogenase | +1.82 | 0.05 | nd | / |
| Inorganic ion transport and metabolism | | | | | |
| AOL_s00079g152 | ATP sulfurylase (sulfate adenylyltransferase) | +2.32 | 0.04 | -2.02 | 0.04 |
| Intracellular trafficking, secretion, and vesicular transport | | | | | |
| AOL_s00079g51 | Vesicle coat complex COPI, beta' subunit | +2.02 | 0.01 | nd | / |
| AOL_s00054g822 | Exocyst subunit - Sec10p | +4.27 | 0 | nd | / |
| AOL_s00110g157 | Vesicle coat complex COPII, subunit SEC24/subunit SFB2 | +2.61 | 0 | nd | / |
| General function prediction only | | | | | |
| AOL_s00080g135 | RNA-binding protein RBM8/Tsunagi (RRM superfamily) | +1.70 | 0.02 | nd | / |
| AOL_s00054g224 | Serine-threonine phosphatase 2B, catalytic subunit | +1.88 | 0 | nd | / |
| AOL_s00076g598 | Dipeptidyl peptidase III | +2.09 | 0 | nd | / |
| AOL_s00006g123 | Predicted GTP-binding protein (ODN superfamily) | -2.59 | 0 | nd | / |
| Others | | | | | |
| AOL_s00078g341 | Function unknown | +4.19 | 0 | nd | / |
| AOL_s00210g113 | Chromosome transmission fidelity protein 18 | +2.14 | 0.02 | nd | / |
| AOL_s00097g14 | NmrA-like family protein | +1.84 | 0.01 | nd | / |
| AOL_s00007g251 | Function unknown | +1.86 | 0.01 | nd | / |
| AOL_s00140g48 | Function unknown | +2.0 | 0 | nd | / |
| AOL_s00080g99 | Function unknown | +3.15 | 0 | nd | / |
| AOL_s00215g807 | Function unknown | +1.93 | 0.05 | nd | / |
| AOL_s00188g77 | Function unknown | -1.70 | 0.01 | nd | / |
| AOL_s00076g128 | Function unknown | +1.94 | 0.01 | nd | / |
| AOL_s00004g593 | Function unknown | -2.14 | 0 | nd | / |
| AOL_s00043g366 | Tyrosinase | -2.14 | 0 | nd | / |
| AOL_s00054g828 | Function unknown | +2.68 | 0 | nd | / |
| AOL_s00188g77 | Function unknown | -1.70 | 0.01 | nd | / |

+, up-regulated; -, down-regulated; ns: significant fluctuation among repeats in this treatment；nd: not detected or not determined; nc: without significant change.
