# Supplementary material for: Genomic and Proteomic Analyses of the Fungus Arthrobotrys oligospora Provide Insights into Nematode-Trap Formation
Source: PLoS Pathog. 2011 Sep 1;7(9):e1002179. doi: 10.1371/journal.ppat.1002179 (PMC3164635; doi:10.1371/journal.ppat.1002179)
Supplement: Table S8 — List of cytochrome P450 from A. oligospora. (DOC) [file ppat.1002179.s013.doc]

**Table S8. List of cytochrome P450 from *A. oligospora*.**

| No. | Gene | P450 Class Name | P450 Cluster Name | Putative CYP Name |
| --- | --- | --- | --- | --- |
| 1 | AOL_s00079g225 | [E-class P450, group I](javascript:openWin('/class.php?a=dv_intro&id=4')) | Cluster 3 | CYP684 |
| 2 | AOL_s00097g384 | [E-class P450, group I](javascript:openWin('/class.php?a=dv_intro&id=4')) | [Cluster 3](http://p450.riceblast.snu.ac.kr/cluster.php?a=dv&id=3) | CYP684 |
| 3 | AOL_s00079g255 | [E-class P450, group I](javascript:openWin('/class.php?a=dv_intro&id=4')) | [Cluster 3](http://p450.riceblast.snu.ac.kr/cluster.php?a=dv&id=3) | CYP628 |
| 4 | AOL_s00080g313 | [E-class P450, group I](javascript:openWin('/class.php?a=dv_intro&id=4')) | [Cluster 3](http://p450.riceblast.snu.ac.kr/cluster.php?a=dv&id=3) | CYP680 |
| 5 | AOL_s00097g277 | [E-class P450, group I](javascript:openWin('/class.php?a=dv_intro&id=4')) | [Cluster 3](http://p450.riceblast.snu.ac.kr/cluster.php?a=dv&id=12) | CYP680 |
| 6 | AOL_s00210g57 | [E-class P450, group I](javascript:openWin('/class.php?a=dv_intro&id=4')) | [Cluster 3](http://p450.riceblast.snu.ac.kr/cluster.php?a=dv&id=3) | CYP680 |
| 7 | AOL_s00215g280 | [E-class P450, group I](javascript:openWin('/class.php?a=dv_intro&id=4')) | [Cluster 3](http://p450.riceblast.snu.ac.kr/cluster.php?a=dv&id=3) | CYP680 |
| 8 | AOL_s00215g22 | [E-class P450, group I](javascript:openWin('/class.php?a=dv_intro&id=4')) | [Cluster 3](http://p450.riceblast.snu.ac.kr/cluster.php?a=dv&id=3) | CYP537 |
| 9 | AOL_s00215g21 | [E-class P450, group I](javascript:openWin('/class.php?a=dv_intro&id=4')) | [Cluster 3](http://p450.riceblast.snu.ac.kr/cluster.php?a=dv&id=3) | CYP680 |
| 10 | AOL_s00007g279 | [E-class P450, group I](javascript:openWin('/class.php?a=dv_intro&id=4')) | [Cluster 5](http://p450.riceblast.snu.ac.kr/cluster.php?a=dv&id=5) | CYP59 |
| 11 | AOL_s00076g486 | [E-class P450, group I](javascript:openWin('/class.php?a=dv_intro&id=4')) | [Cluster 6](http://p450.riceblast.snu.ac.kr/cluster.php?a=dv&id=6) | CYP544 |
| 12 | AOL_s00210g299 | [E-class P450, group I](javascript:openWin('/class.php?a=dv_intro&id=4')) | [Cluster 6](http://p450.riceblast.snu.ac.kr/cluster.php?a=dv&id=6) | CYP617 |
| 13 | AOL_s00075g118 | [E-class P450, group I](javascript:openWin('/class.php?a=dv_intro&id=4')) | [Cluster 7](http://p450.riceblast.snu.ac.kr/cluster.php?a=dv&id=7) | CYP620 |
| 14 | AOL_s00078g505 | [E-class P450, group I](javascript:openWin('/class.php?a=dv_intro&id=4')) | [Cluster 7](http://p450.riceblast.snu.ac.kr/cluster.php?a=dv&id=7) | CYP620 |
| 15 | AOL_s00081g120 | [E-class P450, group I](javascript:openWin('/class.php?a=dv_intro&id=4')) | [Cluster 7](http://p450.riceblast.snu.ac.kr/cluster.php?a=dv&id=7) | CYP620 |
| 16 | AOL_s00176g104 | [E-class P450, group I](javascript:openWin('/class.php?a=dv_intro&id=4')) | [Cluster 7](http://p450.riceblast.snu.ac.kr/cluster.php?a=dv&id=7) | CYP620 |
| 17 | AOL_s00078g25 | [E-class P450, group I](javascript:openWin('/class.php?a=dv_intro&id=4')) | [Cluster 7](http://p450.riceblast.snu.ac.kr/cluster.php?a=dv&id=7) | CYP530 |
| 18 | AOL_s00215g282 | [E-class P450, group I](javascript:openWin('/class.php?a=dv_intro&id=4')) | [Cluster 7](http://p450.riceblast.snu.ac.kr/cluster.php?a=dv&id=7) | CYP619 |
| 19 | AOL_s00097g233 | [E-class P450, group I](javascript:openWin('/class.php?a=dv_intro&id=4')) | [Cluster 12](http://p450.riceblast.snu.ac.kr/cluster.php?a=dv&id=12) | CYP5095 |
| 20 | AOL_s00006g325 | [E-class P450, group I](javascript:openWin('/class.php?a=dv_intro&id=4')) | [Cluster 19](http://p450.riceblast.snu.ac.kr/cluster.php?a=dv&id=19) | CYP505 |
| 21 | AOL_s00043g740 | [E-class P450, group I](javascript:openWin('/class.php?a=dv_intro&id=4')) | [Cluster 35](http://p450.riceblast.snu.ac.kr/cluster.php?a=dv&id=5) | CYP534 |
| 22 | AOL_s00215g278 | [E-class P450, group I](javascript:openWin('/class.php?a=dv_intro&id=4')) | [Cluster 35](http://p450.riceblast.snu.ac.kr/cluster.php?a=dv&id=35) | CYP666 |
| 23 | AOL_s00004g577 | [E-class P450, group IV](javascript:openWin('/class.php?a=dv_intro&id=6')) | [Cluster 9](http://p450.riceblast.snu.ac.kr/cluster.php?a=dv&id=9) | CYP512 |
| 24 | AOL_s00076g401 | [E-class P450, group IV](javascript:openWin('/class.php?a=dv_intro&id=4')) | [Cluster 9](http://p450.riceblast.snu.ac.kr/cluster.php?a=dv&id=9) | CYP512 |
| 25 | AOL_s00173g76 | [E-class P450, group IV](javascript:openWin('/class.php?a=dv_intro&id=6')) | [Cluster 9](http://p450.riceblast.snu.ac.kr/cluster.php?a=dv&id=9) | CYP559 |
| 26 | AOL_s00076g231 | [E-class P450, group IV](javascript:openWin('/class.php?a=dv_intro&id=6')) | [Cluster 13](http://p450.riceblast.snu.ac.kr/cluster.php?a=dv&id=13) | CYP51 |
| 27 | AOL_s00080g314 | [E-class P450, group IV](javascript:openWin('/class.php?a=dv_intro&id=6')) | [Cluster 13](http://p450.riceblast.snu.ac.kr/cluster.php?a=dv&id=13) | CYP51 |
| 28 | AOL_s00109g44 | [E-class P450, group IV](javascript:openWin('/class.php?a=dv_intro&id=6')) | [Cluster 15](http://p450.riceblast.snu.ac.kr/cluster.php?a=dv&id=15) | CYP61 |
| 29 | AOL_s00054g74 | [E-class P450, group IV](javascript:openWin('/class.php?a=dv_intro&id=6')) | [Cluster 20](http://p450.riceblast.snu.ac.kr/cluster.php?a=dv&id=20) | CYP637 |
| 30 | AOL_s00076g400 | [E-class P450, group IV](javascript:openWin('/class.php?a=dv_intro&id=6')) | [Cluster 43](http://p450.riceblast.snu.ac.kr/cluster.php?a=dv&id=43) | CYP639 |
| 31 | AOL_s00078g599 | [E-class P450, group IV](javascript:openWin('/class.php?a=dv_intro&id=6')) | [Cluster 58](http://p450.riceblast.snu.ac.kr/cluster.php?a=dv&id=58) | CYP5157 |
| 32 | AOL_s00043g381 | [Pisatin demethylase-like](javascript:openWin('/class.php?a=dv_intro&id=16')) | [Cluster 3](http://p450.riceblast.snu.ac.kr/cluster.php?a=dv&id=3) | CYP548 |
| 33 | AOL_s00097g274 | [Pisatin demethylase-like](javascript:openWin('/class.php?a=dv_intro&id=16')) | [Cluster 3](http://p450.riceblast.snu.ac.kr/cluster.php?a=dv&id=3) | CYP548 |
| 34 | AOL_s00078g577 | [Pisatin demethylase-like](javascript:openWin('/class.php?a=dv_intro&id=16')) | [Cluster 3](http://p450.riceblast.snu.ac.kr/cluster.php?a=dv&id=3) | CYP532 |
| 35 | AOL_s00110g222 | [Pisatin demethylase-like](javascript:openWin('/class.php?a=dv_intro&id=16')) | [Cluster 3](http://p450.riceblast.snu.ac.kr/cluster.php?a=dv&id=3) | CYP532 |
| 36 | AOL_s00109g132 | [P450, CYP52](javascript:openWin('/class.php?a=dv_intro&id=8')) | [Cluster 6](http://p450.riceblast.snu.ac.kr/cluster.php?a=dv&id=6) | CYP52 |
