# Supplementary material for: Genomic and Proteomic Analyses of the Fungus Arthrobotrys oligospora Provide Insights into Nematode-Trap Formation
Source: PLoS Pathog. 2011 Sep 1;7(9):e1002179. doi: 10.1371/journal.ppat.1002179 (PMC3164635; doi:10.1371/journal.ppat.1002179)
Supplement: Table S9 — Putative genes coding for peroxisomal proteins in the A. oligospora genome. (DOC) [file ppat.1002179.s014.doc]

**Table S9. Partial list of putative genes coding for peroxisomal proteins in the *A. oligospora* genome.**

| Gene ID | Length (aa) | Annotation |
| --- | --- | --- |
| AOL_s00054g771 | 1194 | Peroxin-1 |
| AOL_s00080g243 | 500 | Peroxin-2 |
| AOL_s00004g97 | 527 | Peroxin-3 |
| AOL_s00215g610 | 454 | Peroxin-3 |
| AOL_s00007g10 | 509 | Peroxin-3 |
| AOL_s00043g169 | 498 | Peroxin-3 |
| AOL_s00054g139 | 471 | Peroxin-3 |
| AOL_s00054g476 | 484 | Peroxin-3 |
| AOL_s00054g767 | 505 | Peroxin-3 |
| AOL_s00054g955 | 529 | Peroxin-3 |
| AOL_s00083g176 | 489 | Peroxin-3 |
| AOL_s00097g504 | 488 | Peroxin-3 |
| AOL_s00097g606 | 464 | Peroxin-3 |
| AOL_s00173g358 | 468 | Peroxin-3 |
| AOL_s00215g652 | 514 | Peroxin-3 |
| AOL_s00075g9 | 162 | peroxin-3 |
| AOL_s00043g671 | 610 | Peroxin-5 |
| AOL_s00043g697 | 1183 | Peroxin-6 |
| AOL_s00176g109 | 363 | Peroxin-7 |
| AOL_s00043g491 | 658 | Peroxin-8 |
| AOL_s00054g861 | 352 | Peroxin-10 |
| AOL_s00083g431 | 366 | Peroxin-11B |
| AOL_s00215g40 | 237 | Peroxin-11B |
| AOL_s00054g410 | 303 | Peroxin-11C |
| AOL_s00006g403 | 1032 | Peroxin-12 |
| AOL_s00004g547 | 395 | Peroxin-12 |
| AOL_s00054g525 | 437 | Peroxin-13 |
| AOL_s00004g292 | 339 | Peroxin-14 |
| AOL_s00081g263 | 456 | Peroxin 14/17 |
| AOL_s00007g540 | 373 | Peroxin-16 |
| AOL_s00169g11 | 828 | Peroxin-16 |
| AOL_s00188g3 | 376 | Peroxin-19 |
| AOL_s00076g677 | 294 | Peroxin-20 |
| AOL_s00075g133 | 622 | Peroxin Pex23-like |
| AOL_s00004g384 | 515 | Peroxin 24 |
| AOL_s00043g516 | 439 | Peroxin 26 |
| AOL_s00097g527 | 561 | Peroxin 31 |
| AOL_s00054g29 | 901 | Peroxisomal multifunctional beta-oxidation protein |
| AOL_s00076g393 | 313 | Peroxisomal multifunctional beta-oxidation protein |
| AOL_s00081g60 | 142 | Peroxisomal 2,4- dienoyl-CoA reductase |
| AOL_s00078g492 | 312 | Peroxisomal 2,4-dienoyl-CoA reductase |
| AOL_s00080g252 | 275 | Peroxisomal 2,4-dienoyl-CoA reductase |
| AOL_s00043g730 | 270 | Peroxisomal D3,D2-enoyl-CoA isomerase |
| AOL_s00054g149 | 425 | Acetyl-CoA C-acyltransferase, peroxisomal |
| AOL_s00078g38 | 418 | 3-ketoacyl-coA thiolase peroxisomal A precursor |
| AOL_s00004g606 | 851 | Peroxisomal ABC transporter (PXA1), putative |
| AOL_s00007g211 | 752 | Peroxisomal AMP binding enzyme, putative |
| AOL_s00043g804 | 169 | Peroxiredoxin |
| AOL_s00043g607 | 476 | Peroxisomal adenine nucleotide transporter |
| AOL_s00078g269 | 216 | Peroxisomal membrane protein 4 |
| AOL_s00079g354 | 697 | Peroxisomal copper amine oxidase |
| AOL_s00083g158 | 324 | Peroxisomal carrier protein, putative |
| AOL_s00215g205 | 306 | Peroxisomal membrane protein Pmp47, putative |
| AOL_s00215g316 | 173 | Protein Mpv17 |
