# Supplementary material for: Genomic and Proteomic Analyses of the Fungus Arthrobotrys oligospora Provide Insights into Nematode-Trap Formation
Source: PLoS Pathog. 2011 Sep 1;7(9):e1002179. doi: 10.1371/journal.ppat.1002179 (PMC3164635; doi:10.1371/journal.ppat.1002179)
Supplement: Table S10 — Putative genes coding for lectins and adhesive proteins in the A. oligospora genome. (DOC) [file ppat.1002179.s015.doc]

**Table S10. Putative genes coding for lectins and adhesive proteins in the *A. oligospora*** genome.

| Gene ID | Length (aa) | Annotation |
| --- | --- | --- |
| Putative genes coding for lectins | | |
| AOL_s00006g511 | 118 | Mannose-specific lectin precursor, putative |
| AOL_s00043g401 | 1428 | Legume lectin beta domain protein |
| AOL_s00043g494 | 329 | Lectin family integral membrane protein, putative |
| AOL_s00043g531 | 480 | Lectin family integral membrane protein, putative |
| AOL_s00076g540 | 343 | Fucose-specific lectin FleA |
| AOL_s00080g288 | 518 | Lectin [*Arthrobotrys oligospora*] |
| AOL_s00083g511 | 756 | Jacalin-like lectin domain-containing protein |
| Putative genes coding for adhesive proteins | | |
| AOL_s00076g567 | 718 | Adhesin protein Mad1 [*Metarhizium anisopliae*] |
| AOL_s00080g55 | 322 | Adhesion regulating molecule, putative |
| AOL_s00215g185 | 1102 | GPI-anchored cell surface glycoprotein |
| AOL_s00215g436 | 964 | Glycoprotein |
| AOL_s00054g571 | 418 | Protein transport membrane glycoprotein Sec20, putative |
| AOL_s00081g40 | 727 | Fasciclin domain family protein |
| AOL_s00097g409 | 390 | Fasciclin |
| AOL_s00004g12 | 385 | Cell surface protein Mas1 |
| AOL_s00043g339 | 466 | Glycolipid anchored surface protein 4 precursor |
| AOL_s00079g492 | 445 | Spherulin 4-like cell surface protein |
| AOL_s00079g500 | 412 | Cell surface protein Mas1, putative |
| AOL_s00080g410 | 516 | Spherulin 4-like cell surface protein |
| AOL_s00083g63 | 257 | Cell surface protein Mas1 |
| AOL_s00043g50 | 453 | Bystin |
| AOL_s00076g207 | 551 | Collagen adhesion protein |
| AOL_s00210g231 | 155 | Carcinoembryonic antigen-related cell adhesion molecule |
| AOL_s00007g5 | 337 | Putative cell agglutination protein |
