# Supplementary material for: Genomic and Proteomic Analyses of the Fungus Arthrobotrys oligospora Provide Insights into Nematode-Trap Formation
Source: PLoS Pathog. 2011 Sep 1;7(9):e1002179. doi: 10.1371/journal.ppat.1002179 (PMC3164635; doi:10.1371/journal.ppat.1002179)
Supplement: Text S1 — Supporting text. (DOC) [file ppat.1002179.s016.doc]

**Supplemental materials and methods**

**Strain and growth conditions**

*Arthrobotrys oligospora* Fresenius (ATCC 24927) was maintained on corn meal agar (CMA) at 26ºC, adjusted to pH 7 using 1 M NaOH for conidia formation. About 2 x 10^4^ conidia were inoculated into 500-ml Erlenmeyer flasks containing 200-ml PL-4 liquid medium [1] and incubated at 26°C, at 180 rpm for 6 days for hyphal growth.

**Isolation of genomic DNA**

Genomic DNA of *A. oligospora* was extracted using a modified protocol of Murray and Thompson [2]. Mycelia of *A. oligospora* were first grounded to a fine powder with liquid nitrogen prior to extraction. The powder was gently dispersed in extraction buffer [1.4 M NaCl, 2% CTAB, 100 mM Tris-HCl (pH 8.0), 20 mM EDTA, and 1% 2-mercaptoethanol] with ratio of 10 ml buffer per gram of mycelia. The mixture was incubated for 30 min at 60°C with occasional gentle mixing. The extract was emulsified by gentle inversion with an equal volume of chloroform/isoamyl alcohol (24:1). After centrifugation (12,000 x g, 10 min), the aqueous phase was removed with a large bore pipette. One-tenth volume of 10% CTAB and 1 M NaCl was added and incubated for 10 min at 60°C. The chloroform/isoamyl alcohol treatment was repeated. RNase was added to the final concentration of 100 μg/ml and incubated in a 65°C water bath for approximately 30 min. An equal volume of phenol/chloroform/isoamyl alcohol (25:24:1) was added and gently emulsified. After centrifugation (12,000 g, 10 min), the aqueous phase was removed with a large bore pipette. The aqueous phase was emulsified by gentle inversion with chloroform/isoamyl alcohol (24:1) and centrifuged. One-tenth volume of 3 M NaAc (pH 5.5) and 2.5 volume absolute ethanol was added and mixed well. The solution was left in a freezer (-80°C) for at least one hour and then spun at 14,000 g at 4°C for 30 min. The supernatant was drained and the pellet was washed with 70% v/v ethanol. After pouring off the ethanol and dried, the DNA was dissolved in TE buffer.

**Genome sequencing, assembly, prediction and analysis**

Sequencing and assembly: High molecular weight DNA was used to construct plasmid libraries with insert size of 4-5 kb and fosmid libraries with insert size of 35 kb. In total, 78 Mb high quality paired-end data was generated by AB 3730 Sanger sequencing platform from plasmid and fosmid libraries. In addition, 1009 MB pyrosequencing data with average read length of 345 bp and 375 Mb pyrosequencing data with average read length of 242 bp were obtained using the Roche 454 Genome Sequencer Titanium/FLX platforms. Pyrosequencing reads and Sanger sequencing reads were assembled by gsAssembler [3] with default parameter settings. Resequencing of low quality regions and closing of gaps were performed by walking on plasmid and fosmid clones and by PCR using custom primers designed by Consed [4].

Repetitive sequences in the genome assembly were identified by searching Repbase using RepeatMasker [5] with default parameter settings, and by *de novo* repetitive sequence search using RepeatModeler with default parameter settings (http://www.repeatmasker.org/RepeatModeler.html).

Gene prediction and annotation: *Ab initio* gene prediction was performed on the genome assembly by Augustus [6], GlimmerHMM [7], and SNAP [8] trained with transcript sequences of *A. oligospora* from this study, and by GeneMark [9] formulated for fungal genomes. A final set of gene models were selected by EvidenceModeler [10] with weight for evidence from each gene prediction software determined by comparing *ab initio* predicted genes with transcript sequences of *A. oligospora* using Exonerate [11], and with transcript alignment supports from using Exonerate to align transcript sequences of *A. oligospora* and other fungal species downloaded from NCBI and TGI [12]. Predicted genes were annotated by BLASTP [13] searches against protein databases with E-value 1e-10: NR (www.ncbi.nlm.nih.gov), KOGs and COGs [14], KEGG [15], UniRef100 [16], STRING [17], and by InterProScan [18] searches against protein domain databases with default parameter settings: Pfam [19], PRINTS [20], SMART [21], PROSITE [22], TigrPfam [23], PANTHER [24]. Pathway mapping was conducted by associating EC assignment and KO assignment with KEGG metabolic pathways based on BLASTP search result. Multiple gene families were constructed by searching each annotated gene against all other genes using BLASTP. The matches with E≤1e-5 and at least 30% sequence identity over 60% of both gene lengths were used for clustering genes based on single linkage transitive closure [25]. Transmembrane domains identification by TMHMM [26] and signal peptide regions identification by SignalP [27] were performed using the InterProScan package with default parameter settings. Synteny analysis was conducted by Spines (Broad Institute) with default parameter settings and by joining syntenic regions separated by intervals less than 50 kb.

Non-coding RNAs: tRNAs were predicted by tRNAscan [28] using default parameter settings. Ribosomal RNAs were identified by BLASTN search with E-value 1e-5 with known rRNA modules of other fungal genomes. Other non-coding RNAs, including snRNAs and miRNAs, were predicted by searching the Rfam database [29] by Infernal ([http://infernal.janelia.org](http://infernal.janelia.org/)) using default parameter settings.

Transcript sequencing and analysis Vegetative hyphae for transcript sequencing was prepared by incubating in 200 ml liquid medium consisting 2 g tryptone and 2 g glucose, at 28°C 180 rpm for 3 days. Transcript sequences assembled by Velvet [30] with K-mer set to 25 were used to evaluate the completeness of the genome assembly by BLASTN with E-value 1E-5. PASA [31] was used with default parameter settings to align transcript sequences to the genome sequence and resulting gene structures were used to train *ab initio* gene prediction software.

**Orthology and phylogenomic analysis**

Predicted proteins in *A. oligospora* were compared with the predicted proteins of ten sequenced fungal genomes. All proteins were searched against all other proteins in these genomes using BLASTP. The matches with E≤1e-5 and at least 30% sequence identity [32] over 60% of both protein lengths [25] were taken as homologous sequences. Bidirectional best hits (BBHs) [33] from the homologous sequences were taken as orthologous sequences. A total of 529 orthologous proteins were obtained and concatenated to infer the phylogenomic relationships among these taxa with PHYLIP [34] using different methods, including Neighbor-joining (NJ), Maximum parsimony (MP) and Maximum likelihood (ML).

**Multiple gene family analysis**

Multigene families were constructed from the homologous sequences based on single linkage transitive closure [25]. A total of 2882 of the 11479 genes were clustering into 789 multigene families. Several gene families related to fungal pathogenicity were manually selected based on previous knowledge and gene annotations, and then expanded via multigene families. In addition, pathogenicity-related genes were also predicted by a whole genome blast analysis against the PHI gene database [35].

**Evolution analysis of pathogenicity-related gene families**

The pathogenicity-related gene families of subtilisin, NRPS and PKS were selected to perform phylogenetic analysis. NJ tree was obtained using MEGA 4.1 [36] using bootstrap analysis with 1,000 replicates. MP tree was constructed using PAUP*4.0b8 [37] with a heuristic search (initial trees were obtained by 100 replicates with random addition and branch swapping with the TBR algorithm). Non-parameter bootstrap (1,000 replicates) was performed to assess the support level for each node on the MP trees. ML tree was obtained using PHYML version 3.0 [38]. Bootstrap analysis with 100 replicates was applied.

**Repeat-induced point mutation (RIP) analysis**

The RIP indices, TpA/ApT and (CpA+TpG)/(ApC+GpT), were determined to detect RIP relics [39,40,41]. The AT content and RIP indices in all sequences was calculated. Windows of 500-bp and 200-bp with 100-bp shifts were performed separately, for the whole genome as well as individually for the coding regions, non-coding regions, exons, introns, multigene families, and repetitive sequences. RIP regions were detected in the 200-bp windows with 100-bp shifts with TpA/ApT ≥ 0.89 and (CpA+TpG)/(ApC+GpT) ≤ 1.03.

**Quantitative PCR analysis**

Total RNA was extracted with Trizol Reagent (Invitrogen, Carlsbad, California), purified with a RNAse Mini Kit (Qiagen, Valencia, Califonia), and then reverse transcribed with PrimeScript RT reagent Kit (Takara Bio Inc., Shiga, Japan) by following the manufacturer’s instructions. Quantitative real-time PCR was conducted with 2 μl reverse transcribed product in a 7300 Real-Time PCR system (Applied Biosystems, California, USA) using Power SYBR Green PCR Master Mix (Applied Biosystems). 18S rDNA gene was used as the internal control. Fold changes were calculated using the formula 2^-(ΔΔCt)^, where ΔΔCt is ΔCt (treatment)-ΔCt (control), ΔCt is Ct (target gene) - Ct (18S), and Ct is the threshold cycle (User's Manual for ABI 7300 Real-Time PCR System).

**Preparation of the *Caenorhabiditis elegans* extract and induction of trap formation**

*Caenorhabiditis elegans* (strain N2) were grown in oatmeal medium at room temperature (ranging from 21ºC to 26ºC) for 6-7 days, washed with deionized water by centrifugation at 8,000 x g for 20 min at 4ºC for three times, and then resuspended in deionized water. Crude *C. elegans* extract was obtained by ultrasonication for 15 min and centrifugation at 10,000 x g for 30 min at 4°C. About 30 ml extract was obtained from 10 g *C. elegans* (wet weight). The extract was sterilized by passing through a 0.22 μm membrane and then stored at -80°C prior to use.

*A. oligospora* was cultured in the liquid PL-4 medium for 6 days. The vegetative hyphae were harvested by filtration through sterile cheesecloth, washed with sterile deionized water for three times, and then induced using the diluted *C. elegans* extract (10%, v/v) in 88-mm petri dishes at 26°C. In each petri dish, about 200 mg hyphae were incubated in the dilute *C. elegans* extract (40 ml). Hyphae incubated with an equal volume of sterile deionized water at the same condition were considered as a negative control. After induction, hyphae were collected by filtration through sterile cheesecloth, washed with sterile deionized water three times, and frozen immediately in liquid nitrogen and maintained at -80ºC prior to use.

**Trap number and fungus-nematode interaction**

For determination of trap number, samples of 1 ml were taken form the culture dishes and beat upon to disperse the traps. The trap samples were dilute into 10 ml. Portions of 1 ml were taken from these samples and spread on to a 2% agar surface in 88-mm petri dishes marked with squares (4 mm^2^). The traps were counted in the light microscope (20 x magnification, Olympus, Japan).

Samples of 1 ml of trap solution formed in the dilute *C. elegans* extract and the hyphae in the negative controls were spread on a 2% agar surface and about 100 nematodes at different developmental stages were added to the agar surface. At an internal of 4 h, observation was done in the light microscope (20 x magnification, Olympus, Japan). Samples were prepared according to the method described by Veenhuis et al. [42], and observed using a S-3000N scanning electron microscope at 15 kV, at the kunming medical college.

**Protein extraction, two-dimensional gel electrophoresis (2-DE) and image analysis**

To extract cytosolic proteins, approximately 2 g mycelia were ground to a fine powder in liquid nitrogen using a cooled mortar. Mycelium powder was resuspended in 10% (w/v) trichloroacetic acid in acetone, and proteins were allowed to precipitate for 2.5 h at -40ºC. The protein pellet was recovered by centrifugation at 20,200 x g for 30 min, rinsed twice with 90% acetone, air dried, and solubilized in 8 M urea, 2 M thiourea, 4% CHAPS (w/v), 1% dithiothreitol (DTT) (w/v) and 0.8% Bio-lyte 3/10 ampholyte (Bio-Rad Laboratories, Hercules, California) by ultrasonication for 15 min using a UP200S sonicator [43] (Hielscher, Teltow, Germany). Insoluble material was removed by centrifugation at 20,200 x g for 30 min. The supernatant was collected and stored at -80ºC prior to use. Protein concentration was determined by the method of Bradford [44] with bovine serum albumin as the standard.

Approximately 1 mg of protein samples were applied on 17 cm IPG strips, pH 4-7 (Bio-Rad Laboratories) or pH6-11 (GE Healthcare Life Sciences, Piscataway, New Jersey), using the anode Ettan IPGphor Cup Loading Manifold (GE Healthcare Life Sciences). The first dimension (IEF) was performed in the IPGphor Isoelectric Focusing System (GE Healthcare Life Sciences) by stepwise increase of the voltage as follows: 0V-500V for 2 h, 500V for 5 h, 500V-3500V for 3 h and finally 3500V continuing until the total volt-hours reached 54 kVh. After completion of IEF, IPG strips were incubated for 15min in equilibration solution I (50 mM Tris pH6.8, 6 M urea, 30% glycerol, 2% SDS, 2% DTT, trace bromophenol blue) and 15 min in equilibration solution II (solution I with 2.5% iodoacetamide instead of DTT). The second dimension was performed on 12% SDS-PAGE gels using Bio-Rad Protean Plus Dodeca Cell, at 15 mA per gel for first 15 min and then at a constant voltage of 250V, until the dye front reached the bottom of the gel. Low-molecular-weight markers were applied next to the acidic end of the IPG strips. Proteins were visualized by Coomassie Brilliant Blue G-250 (BBI Research, Madison, Wisconsin).

The gel images were acquired by scanning with a UMAX Powerlook 2100XL scanner. Analysis of profiles and statistical analysis of protein spot data were performed with the PDQuest 7.3.0 software (Bio-Rad Laboratories). Gels were normalized based on the total spot integrated volume (area × density) in each gel of the match set. The value assigned to a protein spot was calculated as a percentage of the sum of volumes of all spots detected and multiply a factor of 1,000,000.

**In-gel digestion and matrix assisted laser desorption ionization/time of flight (MALDI-TOF) analysis**

Protein spots were manually excised from Coomassie Brilliant Blue G-250 stained gels. Excised gel spots were first washed with pure water for 15 min and then with 50% (v/v) acetonitrile containing 25 mM NH_4_HCO_3_ three times for 15 min, then again with pure water for 15 min. The gel spots were dehydrated by in 50 μl 100% ACN for 20 min at room temperature and digested in 25 mM NH_4_HCO_3_ containing 5 μg/ml modified trypsin (Promega, Madison, Wisconsin) for 16-20 h at 37ºC to generate peptides.

Peptides (0.3 μl) were spotted on a MALDI sample plate with the same volume of matrix (10 mg/ml α-Cyano-4-hydroxycinnamic acid in 50% acetonitrile, 0.1% trifluoroacetic acid), and analyzed on a 4700 series Proteomics Analyzer (Applied Biosystems). Peptide mass spectra were obtained by averaging 1000 aquired spectra in the positive ion reflector mode with a m/z range of 700 to 4000, and internally calibrated with a mass standard kit for the 4700 Proteomics Analyzer. The 4000 series explorer software 3.0 (Applied Biosystems) was used for peak list generating (S/N>10). Proteins were identified by automated peptide mass fingerprinting using the mascot algorithm of the GPS Explorer 3.5 software (Applied Biosystems) against an in-house sequence database of *A. oligospora*. Positive identifications were accepted up to 95% of confidence level. The following criteria were used for the database searches: at least four matching peptide masses; maximum one missed cleavage per peptide; mass tolerance of 0.1 Da, and the acceptation of carbamidomethylation for cysteine and oxidation for methionine.

Statistical analysis. Samples from three independent experiments were analyzed by 2-DE. Three replicated gels were run for each sample. Spots showing statistically significant difference of at least 1.5-fold (*p* < 0.05) from the three replicated gels were identified as up- or down-regulated proteins.

**Disruption of the gene *p186* in the *A. oligospora* genome**

The *p186* gene was disrupted by homologous recombination according to the method described by Colot et al. [45]. Two fragments (2500-bp and 2800-bp) corresponding to the 5’ and 3’ of the *P186* ORF (5’ and 3’ ﬂanking regions) were ampliﬁed using primers P186-5F/P186-5R and P186-3F/P186-3R, respectively. Primers were designed and synthesized as follows. P186-5F: 5-GTAACGCCAGGGTTTTCCCAGTCACGACGACACGAGTACCCATGCAACA-3; P186-5R: 5-ATCCACTTAACGTTACTGAAATCTCCAACCCCATGGTCACACTCAACAG-3; P186-3F: 5-CTCCTTCAATATCATCTTCTGTCTCCGACGGGCTAACAGATCAGGTCCA-3; P186-3R: 5-GCGGATAACAATTTCACACAGGAAACAGCCCTTTCCTCTGCACCTTCTG-3. **Synthesis of deletion cassette fragments by PCR using primers** P186-5F/P186-3R. Protoplasts of *A. oligospora* were prepared and transformed as described previously [46]. Transformants were selected by incubated on solid regeneration media with 200 µg ml^-1^ hygromycin B, and verified by PCR using primers P186-5F/P186-3R.

**References**

1. Yang JK, Huang XW, Tian BY, Wang M, Niu QH, et al. (2005) Isolation and characterization of a serine protease from the nematophagous fungus *Lecanicillium psalliotae*, displaying nematicidal activity. Biotechnol Lett 27: 1123–1128.

2. Murray MG, Thompson WF (1980) Rapid isolation of high molecular weight plant DNA. Nucleic Acids Res 8: 4321–4325.

3. Margulies M, Egholm M, Altman WE, Attiya S, Bader JS, et al. (2005) Genome sequencing in microfabricated high-density picolitre reactors. Nature 437: 376–380.

4. Gordon D, Desmarais C, Green P (2001) Automated finishing with autofinish. Genome Res 11: 614–625.

5. Jurka J, Kapitonov VV, Pavlicek A, Klonowski P, Kohany O, et al. (2005) Repbase Update, a database of eukaryotic repetitive elements. Cytogent Genome Res 110: 462–467.

6. Stanke M, Waack S (2003) Gene prediction with a hidden Markov model and a new intron submodel. Bioinformatics 19: ii215–ii225.

7. Majoros WH, Pertea M, Salzberg SL (2004) TigrScan and GlimmerHMM: two open source ab initio eukaryotic gene-finders. Bioinformatics 20: 2878–2879.

8. Korf I (2004) Gene finding in novel genomes. BMC Bioinformatics 5: 59.

9. Ter-Hovhannisyan V, Lomsadze A, Chernoff YO, Borodovsky M (2008) Gene prediction in novel fungal genomes using an ab initio algorithm with unsupervised training. Genome Res 18: 1979–1990.

10. Haas BJ, Salzberg SL, Zhu W, Pertea M, Allen JE, et al. (2008) Automated eukaryotic gene structure annotation using EVidenceModeler and the program to assemble spliced alignments. Genome Biol 9: R7.

11. Slater GSC, Birney E (2005) Automated generation of heuristics for biological sequence comparison. BMC Bioinformatics 6: 31.

12. Quackenbush J, Cho J, Lee D, Liang F, Holt I, et al. (2001) The TIGR Gene Indices: analysis of gene transcript sequences in highly sampled eukaryotic species. Nucleic Acids Res 29: 159–164.

13. Altschul SF, Madden TL, Schäffer AA, Zhang J, Zhang Z, et al. (1997) Gapped BLAST and PSI-BLAST: a new generation of protein database search programs. Nucleic Acids Res 25: 3389–3402.

14. Tatusov RL, Fedorova ND, Jackson JD, Jacobs AR, Kiryutin B, et al. (2003) The COG database: an updated version includes eukaryotes. BMC Bioinformatics 4: 41.

15. Kanehisa M, Goto S, Kawashima S, Okuno Y, Hattori M (2004) The KEGG resources for deciphering the genome. Nucleic Acids Res 32: D277–D280.

16. Jensen LJ, Kuhn M, Stark M, Chaffron S, Creevey C, et al. (2009) STRING 8-a global view on proteins and their functional interactions in 630 organisms. Nucleic Acids Res 37: D412–D416.

17. Chang A, Scheer M, Grote A, Schomburg I, Schomburg D (2009) BRENDA, AMENDA and FRENDA the enzyme information system: new content and tools in 2009. Nucleic Acids Res 37: D588–D592.

18. Quevillon E, Silventoinen V, Pillai S, Harte N, Mulder N, et al. (2005) InterProScan: protein domains identifier. Nucleic Acids Res 33: W116–W120.

19. Finn RD, Mistry J, Schuster-Böckler B, Griffiths-Jones S, Hollich V, et al. (2006) Pfam: clans, web tools and services. Nucleic Acids Res 34: D247–D251.

20. Attwood TK, Bradley P, Flower DR, Gaulton A, Maudling N, et al. (2003) PRINTS and its automatic supplement, prePRINTs. Nucleic Acids Res 31: 400–402.

21. Letunic I, Copley RR, Pils B, Pinkert S, Schultz J, et al. (2006) SMART 5: domains in the context of genomes and networks. Nucleic Acids Res 34: D257–D260.

22. Hulo N, Bairoch A, Bulliard V, Cerutti L, De Castro E, et al. (2006) The PROSITE database. Nucleic Acids Res 34: D227–D230.

23. Haft DH, Selengut JD, White O (2003) The TIGRFAMs database of protein families. Nucleic Acids Res 31: 371–373.

24. Thomas PD, Campbell MJ, Kejariwal A, Mi H, Karlak B, et al. (2003) PANTHER: a library of protein families and subfamilies indexed by function. Genome Res 13: 2129–2141.

25. Galagan JE, Calvo SE, Borkovich KA, Selker EU, Read ND, et al. (2003) The genome sequence of the filamentous fungus *Neurospora crassa*. Nature 422: 859–868.

26. Krogh A, Larsson B, von Heijne G, Sonnhammer EL (2001) Predicting transmembrane protein topology with a hidden Markov model: application to complete genomes. J Mol Biol 305: 567–580.

27. Bendtsen JD, Nielsen H, von Heijne G, Brunak S (2004) Improved prediction of signal peptides: SignalP 3.0. J Mol Biol 340: 783–795.

28. Lowe TM, Eddy SR (1997) tRNAscan-SE: a program for improved detection of transfer RNA genes in genomic sequence. Nucleic Acids Res 25: 955–964.

29. Griffiths-Jones S, Moxon S, Marshall M, Khanna A, Eddy SR, et al. (2005) Rfam: annotating non-coding RNAs in complete genomes. Nucleic Acids Res 33: D121–D124.

30. Chaisson MJ, Brinza D, Pevzner PA (2009) De novo fragment assembly with short mate-paired reads: does the read length matter. Genome Res 19: 336–346.

31. Haas BJ, Delcher AL, Mount SM, Wortman JR, Smith RK Jr, et al. (2003) Improving the Arabidopsis genome annotation using maximal transcript alignment assemblies. Nucleic Acids Res 31: 5654–5666.

32. Rost B (1999) Twilight zone of protein sequence alignments. Protein Eng 12: 85–94.

33. Overbeek R, Fonstein M, D'Souza M, Pusch GD, Maltsev N (1999) The use of gene clusters to infer functional coupling. Proc Natl Acad Sci USA 96: 2896–2901.

34. Felsenstein J (1989) PHYLIP-Phylogeny inference package (Version 3.2). Cladistics 5: 164-166.

35. Winnenburg R, Baldwin TK, Urban M, Rawlings C, Kohler J, et al. (2006) PHI-base: a new database for pathogen host interactions. Nucleic Acids Res 34: D459–D464.

36. Tamura K, Dudley J, Nei M, Kumar S (2007) MEGA4: Molecular evolutionary genetics analysis (MEGA) software version 4.0. Mol Biol Evol 24: 1596–1599.

37. Swofford D (2002) PAUP*: phylogenetic analysis using parsimony (*and other methods). Sunderland, MA, USA: Sinauer Associates Inc.

38. Guindon S, Gascuel O (2003) A simple, fast, and accurate algorithm to estimate large phylogenies by maximum likelihood. Syst Biol 52: 696–704.

39. Margolin BS, Garrett-Engele PW, Stevens JN, Fritz DY, Garrett-Engele C, et al. (1998) A methylated *Neurospora* 5S rRNA pseudogene contains a transposable element inactivated by repeat-induced point mutation. Genetics 149:787–97.

40. Hane JK, Oliver RP (2008) RIPCAL: a tool for alignment-based analysis of repeat-induced point mutations in fungal genomic sequences. BMC Bioinformatics 9: 478.

41. Watters MK, Randall TA, Margolin BS, Selker EU, Stadler DR (1999) Action of repeat-induced point mutation on both strands of a duplex and on tandem duplications of various sizes in *Neurospora*. Genetics 153: 705–714.

42. Veenhuis M, Nordbring-Hertz B, Harder W (1985) An electron-microscopical analysis of capture and initial stages of penetration of nematodes by *Arthrobotrys oligospora*. Antonie van Leeuwenhoek 51: 385–398.

43. Fernández-Acero FJ, Jorge I, Calvo E, Vallejo I, Carbú M, et al. (2007) Proteomic analysis of phytopathogenic fungus *Botrytis cinerea* as a potential tool for identifying pathogenicity factors, therapeutic targets and for basic research. Arch Microbiol 187: 207–215.

44. Bradford M (1976) A **rapid** and **sensitive method** for the **quantitation** of **microgram** **quantities** of **protein utilizing** the **principle** of **protein-dye binding**. Anal Biochem 72: 248–254.

45. Colot HV, Park G, Turner GE, Ringelberg C, Crew CM, et al. (2006) A high-throughput gene knockout procedure for *Neurospora* reveals functions for multiple transcription factors. Proc Natl Acad Sci USA 103: 10352–10357.

46. Tunlid A, Åhman J, Oliver RP (1999) Transformation of the nematode-trapping fungus *Arthrobotrys oligospora*. FEMS Microbiol Lett 173: 111-116.
